# Engineering of buried interfaces in perovskites: advancing sustainable photovoltaics

**DOI:** 10.1186/s40580-024-00464-z

**Published:** 2024-12-16

**Authors:** Jihyun Kim, William Jo

**Affiliations:** 1 New and Renewable Energy Research Center, Ewha Womans University, Seoul, 03760 Korea; 2 Department of Physics, Ewha Womans University, Seoul, 03760 Korea

## Abstract

Perovskite solar cells (PSCs) have garnered significant attention for their high power conversion efficiency (PCE) and potential for cost-effective, large-scale manufacturing. This comprehensive review focuses on the role of buried interface engineering in enhancing the performance and stability of PSCs with both n-type electron transport layer/perovskite/p-type hole transport layer (n-i-p) and p-type hole transport layer/perovskite/n-type electron transport layer (p-i-n) structures. This study highlights key challenges associated with interface engineering, such as charge extraction, recombination loss, and energy level alignment. Various interface engineering techniques, such as surface passivation, self-assembled monolayers, and additive engineering, are explored in terms of their effectiveness in mitigating recombination loss and improving long-term device stability. This review also provides an in-depth analysis of material selection for the electron and hole transport layers, defect management techniques, and the influence of these on perovskite film quality and device stability. Advanced characterization methods for buried interfaces are discussed, providing insights into the structural, morphological, and electronic properties that govern device performance. Furthermore, we explore emerging approaches that target homogenous cation distribution and phase stability at buried interfaces, both of which are crucial for improving PCEs beyond current benchmarks. By synthesizing the latest research findings and identifying key challenges, this review aims to guide future directions in interface engineering for PSCs and ensure their successful use in next-generation sustainable energy technologies.

**Graphical Abstract:**

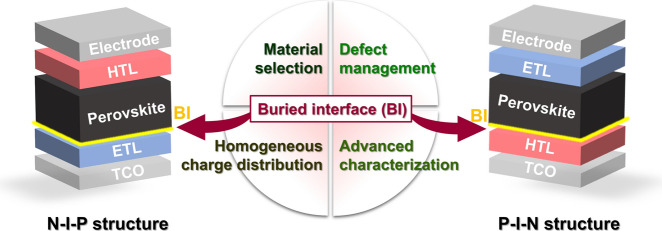

## Introduction

Perovskite solar cells (PSCs) have recently emerged as an important photovoltaic technology over the past decade based on their high efficiency and cost-effective manufacturing potential [1–3]. In particular, their certified power conversion efficiency (PCEs) has increased from 3.8 to 26%, rivaling, and in some cases surpassing, traditional silicon-based photovoltaics [4]. This improvement in performance, coupled with the versatility of solution-based processing and the promise of low-cost, large-scale production, has positioned perovskite photovoltaics as an important component of next-generation sustainable energy applications [5–8]. The excellent performance of PSCs is based on the presence of metal halide perovskites, which are characterized by an ABX_3_ crystal structure, [9] where A is typically an organic cation (e.g., methylammonium, formamidinium [FA]) or an inorganic cation (e.g., cesium), B is a metal cation (usually lead or tin), and X is a halide anion (chloride, bromide, or iodide) [10]. These materials offer excellent optoelectronic properties, including a high absorption coefficient, long carrier diffusion lengths, and a tunable bandgap [11, 12]. Of the various PSC architectures that have been designed and tested, two dominant configurations have emerged: the electron transport layer (ETL)/perovskite/hole transport layer (HTL) structure (n-i-p) and the HTL/perovskite/ETL structure (p-i-n). Although both architectures have achieved excellent performances, opportunities remain for further optimization.

Central to the performance and longevity of PSCs are the buried interfaces between the perovskite absorber layer and the adjacent charge transport layers [13]. These buried interfaces play an important role in determining the overall efficiency and stability of PSCs because charge extraction, carrier recombination, and energy level alignment occur at these interfaces. The quality and characteristics of these interfaces thus influence the open-circuit voltage (V_OC_), short-circuit current density (J_SC_), fill factor (FF), and the PCE of PSCs [14]. In particular, these interfaces are often the initiation points for degradation processes that can compromise the long-term stability of devices [15]. The strategic design and engineering of these buried interfaces have thus become a focus of research designed to narrow the gap between the performance of PSCs and theoretical limits while also enhancing their long-term operational stability [16].

This review paper focuses on recent advances in buried interface engineering for both n-i-p and p-i-n PSCs. In particular, we summarize innovative strategies that have been developed to optimize these junctions, including surface passivation, the implementation of self-assembled monolayers (SAMs), and the use of additives [17, 18]. We examine the diverse array of materials employed as ETLs and HTLs and how their properties influence interface dynamics. Special attention is also given to recent advancements in interface modification techniques and their impact on device performance [19]. A critical examination of the theoretical underpinnings for and practical approaches to the mitigation of defects at buried interfaces, including grain boundary passivation and novel interface engineering methodologies, is presented, and we also explore the influence of interface engineering on the thermal stability, photostability, and long-term operational stability of PSCs, particularly in flexible devices. We discuss innovative approaches designed to ensure a uniform cation distribution and phase stability at buried interfaces, together which enhance device performance and longevity [20, 21]. By providing a comprehensive review of the latest advancements in buried interface engineering for PSCs, this paper offers valuable insights into the current state of the field and highlights promising pathways for future research. The strategic design of buried interfaces in perovskites has emerged as a key driver of highly efficient, stable, and sustainable solar energy solutions. This review can thus contribute to the ongoing dialogue within the scientific community and inspire new directions in the pursuit of perovskite-based photovoltaics that can meet the growing global demand for clean, renewable energy.

## Material composition and buried interface engineering

### Perovskite/ETL buried interfaces in n-i-p structures

In the n-i-p structure, the ETL contributes to both the performance and longevity of PSCs. The primary function of the ETL is to efficiently extract and transport electrons from the perovskite absorber layer to the electrode while simultaneously blocking the movement of holes [22]. This selective charge extraction minimizes recombination loss and maximizes the PCE of the solar cell, [23] thus appropriate ETL materials need to be selected for the design and optimization of high-performance PSCs. Various materials have been explored as ETLs in n-i-p structured PSCs, including metal oxides such as SnO₂, TiO₂, and ZnO and organic compounds such as phenyl-C61-butyric acid methyl ester (PCBM) and fullerene C₆₀ [24, 25]. Of these, SnO₂ and TiO₂ have become the most widely favored choices for high-efficiency devices due to their excellent chemical and thermal stability, which leads to more consistent device performance over long periods and under various environmental conditions [26]. While alternative ETL materials such as ZnO, PCBM, and C₆₀ have shown promise in certain situations, they are often limited in other areas. For example, although ZnO promotes electron mobility, it suffers from chemical instability when in contact with perovskite materials [27]. Similarly, organic ETLs such as PCBM and C₆₀ have a favorable energy level alignment but lower electron mobility and inferior long-term stability compared to their inorganic counterparts [28]. Thus, despite the advancements that have been reported to date, engineering of the buried interfaces in PSCs is required to address intrinsic defects such as oxygen vacancies, optimize energy level alignment for improved charge transfer, and enhance interfacial stability, thus improving overall performance. Interface engineering also reduces charge accumulation and hysteresis while improving the quality of the perovskite film. In addition, as PSC technology shifts towards large-scale production, effective interface engineering is required to maintain performance and stability across larger fabrication areas.

### Perovskite/HTL buried interfaces in p-i-n structures

Inverted PSCs have demonstrated superior PCE and long-term stability compared to conventional structures due to their more favorable band alignment, higher efficiency, improved stability, and compatibility with flexible substrates [29–31]. These advantages are particularly useful for tandem PSCs, in which the inverted architecture enhances overall device performance [32]. A key component of p-i-n PSCs is the HTL, which extracts positive charge carriers (holes) from the perovskite absorber and transfers them to the electrode. The development of p-type materials as HTLs for these inverted PSCs has progressed through several stages, each marked by significant advancements in performance and stability. These improvements reflect ongoing efforts to overcome issues associated with long-term stability and scalable manufacturing in perovskite photovoltaics. Organic conducting polymers such as poly (3,4-ethylenedioxythiophene) polystyrene sulfonate (PEDOT:PSS) were among the first materials used as HTLs [33]. PEDOT: PSS became widespread due to its solution processability, low cost, and good conductivity, making it suitable for simple fabrication at low temperatures [33, 34]. It proved effective for efficient hole extraction and improved the stability of inverted PSCs compared to traditional n-i-p structures [35]. However, the limitations of PEDOT: PSS, particularly its poor long-term stability and interaction with the perovskite layer under humid conditions, prompted researchers to seek alternative materials. This led to the adoption of poly (triarylamine) (PTAA) as a more robust HTL [36, 37]. PTAA offers superior chemical stability, a higher glass transition temperature, and better overall device stability, particularly in humid environments, resulting in higher PCEs [36].

As research progressed, attention shifted towards optimizing the interface between the perovskite layer and the HTL. The introduction of passivation layers and interface engineering techniques were used to reduce interfacial defect density, leading to significant improvements in both efficiency and stability [16]. Other researchers explored inorganic alternatives to organic HTLs, with nickel oxide (NiO_x_) emerging as a promising candidate due to its high stability and favorable energy level alignment with perovskite materials [31, 38]. Current research focuses on developing doped variants of materials such as PEDOT: PSS, PTAA, and NiO_x_, as well as hybrid organic–inorganic HTLs, to further reduce interface losses, improve operational stability, and simplify manufacturing processes for large-scale production. While p-i-n PSCs have exhibited great potential for flexible, roll-to-roll mass production due to their simpler architecture and low-temperature processing, challenges remain in achieving PCEs comparable to n-i-p PSCs. One of the main issues is the significant energy loss at the HTL/perovskite buried interface, primarily caused by energy level mismatches and inefficient charge transfer, which limit the overall performance of p-i-n PSCs despite their manufacturing advantages.

### Tailoring buried interfaces: defect mitigation and grain boundary passivation in ETL and HTL layers

Highly efficient PSCs typically use FAPbI_3_ as the light-absorbing layer, but the size mismatch between FA^+^ and [PbI_6_]^4−^ octahedra leads to inherent instability and spontaneous phase transitions at room temperature [43]. However, the multi-component nature and solution-based preparation of these materials generate abundant intrinsic point defects, including interstitials, vacancies, and antisites [44]. These defects act as recombination centers for charge carriers, severely limiting the PCE of PSCs and accelerating performance degradation in the presence of environmental stressors. Interface-associated non-radiative recombination processes stemming from mismatched energy alignment, interfacial defects, and charge back-transfer reactions also contribute to carrier loss [39]. The ETL in the n-i-p structure, which typically consists of materials such as SnO_2_ and TiO_2_, contains its own set of intrinsic defects, including oxygen vacancies (V_o_) and interstitial atoms, promoting the radiative and non-radiative recombination of carriers at the ETL surface, further compromising device performance [45–47]. Therefore, adopting passivation materials and testing their effectiveness should be considered before employing modifications.

**Fig. 1:**
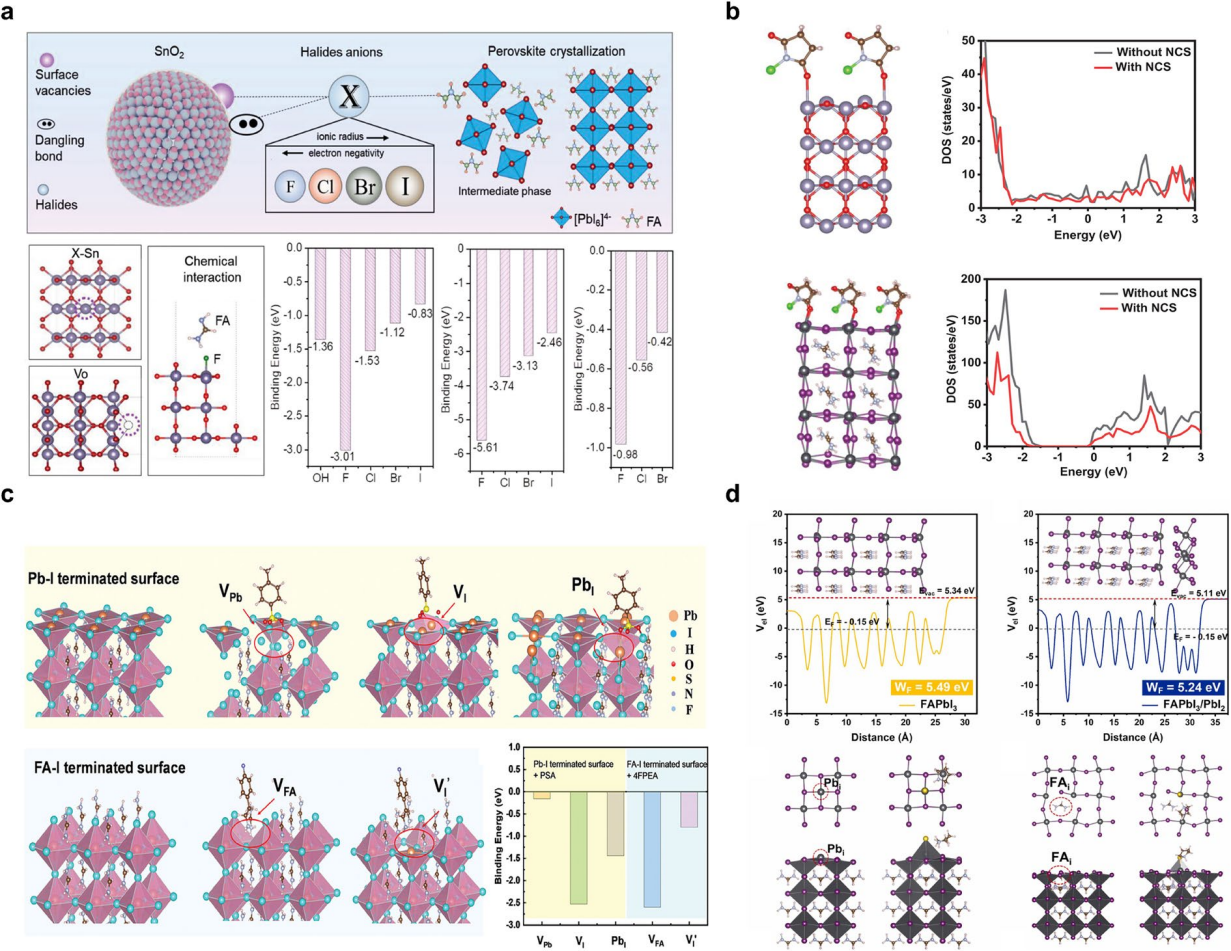
**a** Chemical interaction between various halides (X = F, Cl, Br, I) and the perovskite–SnO₂ interface, including their binding energies at Sn sites and oxygen vacancies, and their hydrogen bonding interactions with FA cations. Reproduced with permission [39]. Copyright 2023, Wiley–VCH GmbH. **b** DFT calculations showing a side view of the N-chlorosuccinimide (NCS)–Sn bonds on the SnO₂ surface and with iodine vacancies (V_I_), along with the density of states for SnO₂ and the perovskite, both with and without modification. Reproduced with permission [40]. Copyright 2024, Wiley–VCH GmbH **c** Schematic diagrams of Pb-I and FA-I terminated surfaces, both with and without defect sites, showing binding interactions with PSA⁻ and 4FPEA⁺. Reproduced with permission [41]. Copyright 2023, Wiley–VCH GmbH. **d** Mechanism underlying the change in the perovskite work function (WF), with the electrostatic potential averaged over planes perpendicular to FAPbI₃ and the FAPbI₃/PbI₂ heterointerface. Reproduced with permission [42]. Copyright 2023, Published by Elsevier Ltd

To understand the role of halide additives in modifying a SnO_2_ ETL and its subsequent impact on perovskite crystallization, Deng et al. [39] conducted a systematic study using the potassium halides KF, KCl, KBr, and KI as additives in the colloidal SnO_2_ precursor (See Fig. 1a). Density functional theory (DFT) simulations were employed to provide atomic-level insights into the interactions between halides and the SnO_2_ surface, focusing on three key components: uncoordinated Sn sites, V_o_, and interactions with FA cations. DFT calculations revealed a clear trend in the binding energy (*E*_*B*_) of halides with uncoordinated Sn sites on the SnO_2_ surface. *E*_*B*_ was highest for F (– 3.01 eV), followed by Cl (– 1.53 eV). Notably, both F and Cl demonstrated stronger binding affinities than the –OH groups (– 1.36 eV) typically present on the SnO_2_ surface. This suggests that F and Cl are particularly effective in passivating surface defects on SnO_2_, potentially leading to improved ETL performance. V_o_ sites are also known to be detrimental to the performance of SnO_2_ as an ETL. The results of simulations showed that F ions exhibit the highest binding energy (– 5.61 eV) for the occupation of V_o_ sites compared with other halides. This strong interaction indicates that F is exceptionally effective in passivating oxygen vacancies, which could lead to reduced trap-assisted recombination at the ETL/perovskite interface. Understanding the interaction between halides and perovskite precursors is crucial for controlling the crystallization dynamics. The DFT results showed that the interaction energy (*E*_*I*_) between FA cations and halides at Sn sites decreased in the order of F > Cl > Br. Interestingly, iodine demonstrated such weak binding that it escaped during FA interaction simulations. This trend suggests that F and Cl play a more significant role in mediating perovskite nucleation and growth on the SnO_2_ surface. Further analysis of FA interactions at V_o_ sites revealed negligible interaction energies for F and Cl, while Br and I had positive values. This indicates that the chemical interaction between halides at V_o_ sites and FA cations is generally weak, with potential implications for the initial stages of perovskite formation.

The efficacy of N-chlorosuccinimide (NCS) as a passivating agent for both a SnO_2_ ETL and the perovskite active layer has been investigated using theoretical calculations and density of states (DOS) analysis [40]. Ding et al. reported significant improvements in defect passivation for both materials, potentially leading to enhanced device performance. It has been established that the surface of SnO_2_ contains a high concentration of dangling bond defects, which can act as recombination centers and hinder charge transport [46]. Theoretical calculations indicated that the C═O bond in NCS can effectively interact with and passivate these surface defects. This interaction is likely due to the strong electron-withdrawing nature of the C═O group, which can form coordinating bonds with unsaturated Sn atoms on the SnO_2_ surface [40]. To quantify the passivation effect, Ding et al. analyzed the DOS for SnO_2_ with and without NCS modification (Fig. 1b). The results revealed a marked reduction in the DOS near the Fermi level (0 eV) for NCS-modified SnO_2_. This decrease in the defect state density was a clear indication of effective defect passivation via a reduction in the number of available trap states that could potentially lead to non-radiative recombination. In addition to its beneficial effects on the SnO_2_ ETL, NCS also demonstrated strong interactions with common defects in the perovskite layer. Calculations revealed that the C═O double bonds in NCS can effectively passivate iodine vacancies (V_I_) and uncoordinated Pb defects in the perovskite structure. These defects are known to be detrimental to device performance, acting as recombination centers and contributing to ion migration. The passivation effect of NCS on perovskite defects was evidenced by the decrease in the DOS for the modified perovskite (Fig. 1b). This reduction in defect states suggests that NCS is capable of mitigating the impact of both surface and bulk defects within the perovskite layer. The dual passivation capability of NCS, which addresses defects in both the SnO_2_ ETL and the perovskite layer, thus represents a promising strategy for improving overall device performance. By simultaneously reducing recombination centers at the ETL/perovskite interface and within the perovskite bulk, NCS modification could potentially lead to enhanced charge extraction, reduced non-radiative recombination, and ultimately, improved PCE in PSCs [40].

To better understand how the amphoteric organic salt 4FPEAPSA affects the perovskite interface, Zhang et al. conducted an in-depth investigation using DFT calculations Fig. 1c and ultraviolet photoelectron spectroscopy (UPS) [41]. Their research employed the Vienna ab initio simulation package (VASP) to perform DFT calculations on Pb-I and FA-I terminated surfaces [48]. They simulated the interaction between 4FPEAPSA and perovskite by pairing the PSA anion with the Pb-I terminated surface and the 4FPEA cation with the FA-I terminated surface. They observed that the S and O atoms in the -SO_3_⁻ group of PSA showed a strong affinity for Pb^2^⁺, leading to crystal structure distortion. Meanwhile, the 4FPEA cation tended to integrate into the [PbX_6_]^4^⁻ octahedral lattice within the FA vacancy (VFA) defect model, potentially enhancing perovskite film crystallization. Binding energy analyses revealed that the 4FPEA cation had a stronger affinity for V_FA_ (− 2.62 eV) compared to V_I_ (− 0.83 eV) on the FA-I terminated surface. On the Pb-I terminated surface, the PSA anion displayed varying binding energies with different defects: − 0.16 eV for V_Pb_, − 2.53 eV for V_I_, and − 1.44 eV for Pb substituted at the I site (PbI). They attributed the stronger binding energy between PSA and V_I_ to newly exposed Pb active sites and coordination interactions [41]. UPS analysis of the hidden perovskite layers revealed significant changes in the energy band configuration after 4FPEAPSA application. The valence band edge shifted upward from − 5.55 eV to − 5.40 eV [41]. Concurrently, the energy difference between the perovskite and MeO-2PACz layers decreased from 0.45 eV to 0.30 eV. This band gap narrowing was expected to reduce the energy barrier at the perovskite-HTL interface, potentially facilitating more efficient hole migration. Furthermore, the Fermi level shifted from − 4.14 eV to − 4.01 eV, aligning with DOS computation predictions. The subtle p-type characteristics observed at the concealed perovskite-HTL interface were found to be beneficial for efficient hole transfer from the perovskite to the MeO-2PACz HTL.

Previous studies have suggested that excess PbI_2_ is randomly dispersed throughout traditional perovskite films such as MAPbI_3_ [49, 50]. For FAPbI_3_-based perovskites, the larger FA^+^ ions cannot readily intercalate into the PbX_6_ framework during the two-step fabrication process, potentially leaving residual PbI_2_ at the buried interface [51, 52]. This excess PbI_2_ in FAPbI_3_ films has been the subject of intense research because it can have both a positive and negative impact on device performance. While excess PbI_2_ can passivate defects and improve device efficiency, it can also lead to stability issues and performance degradation over time. To understand the mechanisms underlying the electronic structure of a reference film and the de-doping process, Ma et al. conducted DFT calculations focusing on the perovskite (PVSK)/PbI_2_ heterointerface [42]. For simplicity, a single-cation FAPbI_3_ perovskite with a (001) PbI-terminated surface in contact with PbI_2_ was examined. The calculations revealed a significant reduction in the WF of the perovskite from 5.49 eV to 5.24 eV upon contact with PbI_2_ (Fig. 1d). This reduction suggested that the FAPbI_3_/PbI_2_ heterointerface led to a decrease in the WF of the perovskite.

Earlier research has reported that dipole moments at the PVSK/PbI_2_ heterointerface can alter the vacuum energy level (E_vac_) of electrons, changing the WF of the perovskite [53–55]. To investigate the mechanisms underlying this reduced WF, the charge-density difference of the FAPbI_3_/PbI_2_ heterointerface has been calculated, revealing that the change in the WF (ΔWF) of the perovskite induced by the FAPbI_3_/PbI_2_ heterointerface has two main causes: charge-density displacement at the FAPbI_3_/PbI_2_ interface and the interfacial interaction-induced charge-density displacement of PbI_2_. This charge-density displacement generates interfacial dipoles, which directly influence the WF of the perovskite. The dipole direction at the FAPbI_3_/PbI_2_ heterointerface points from FAPbI_3_ to PbI_2_. These dipoles shift the local E_vac_ downwards, consequently decreasing the WF of the perovskite [56]. Importantly, at both the perovskite surface and the buried interface where PbI_2_ is enriched, more FAPbI_3_/PbI_2_ heterointerfaces form, leading to a further reduction in the WF and an undesirable energy band structure. Typically, there are two approaches to modifying the WF of a perovskite. The first involves creating an interface dipole by tuning the charge transfer within the heterostructure, [57, 58] while the second employs defect doping, which changes the dominant defect type within the perovskite [59–61]. Notably, theoretical predictions suggest that these trap states can be effectively removed using PyI passivation [42]. This provides a clear pathway for improving the electronic properties of perovskite films through targeted defect passivation.

In conclusion, DFT calculations have been employed to gain valuable insights into the mechanisms underlying modifications to the electronic structure at the interface between the perovskite and charge transport layers. By understanding these processes, more effective strategies for interface engineering and defect passivation can be developed, ultimately improving the performance of perovskite-based devices.

To mitigate the effects of the buried interface at the ETL/PVSK junction in n-i-p structures and the HTL/PVSK interface in p-i-n structures, a range of advanced engineering strategies has been developed. In n-i-p structures, key approaches include the surface modification of the ETL using SAMs or ultrathin interfacial layers to enhance energy-level alignment and to minimize recombination [66]. Additionally, ETL doping with specific dopants has been employed to improve conductivity and adjust the WF of the ETL, [67, 68] resulting in more efficient charge extraction. Passivation techniques are also used, introducing passivation materials to minimize defects at the interface and suppress non-radiative recombination [69, 70]. Similarly, for the HTL/perovskite interface in p-i-n structures, various molecular engineering approaches have been employed. These include the design and synthesis of novel HTL materials with optimized energy levels and improved charge transfer properties, as well as the incorporation of additives to enhance hole conductivity and refine the interaction between the HTL and the perovskite layers [31, 41, 64, 71–73]. Additionally, interface modification techniques, such as applying thin buffer layers or surface treatments to the HTL, can be used to optimize the surface coverage, enhance hole extraction, reduce interfacial defects, and improve the wettability of the HTL [65, 74–77].

**Fig. 2:**
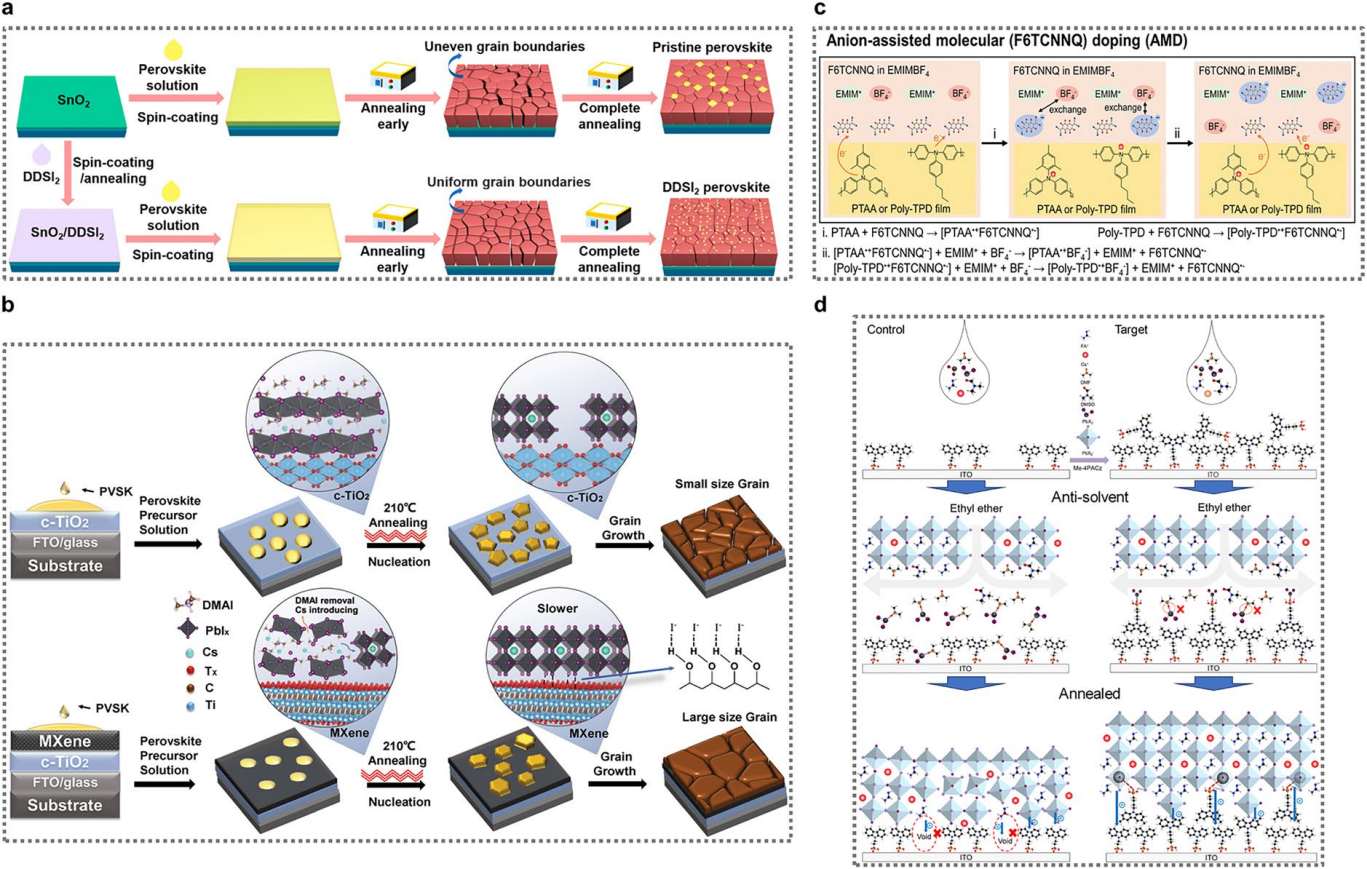
**a** Schematic illustration of the fabrication steps for perovskite films with and without DDSI₂. Reproduced with permission [62]. Copyright 2022, American Chemical Society. **b** Illustrations depicting the nucleation and growth processes of perovskite films with and without MXene as the buried bottom interface. Reproduced with permission [63]. Copyright 2023, Wiley–VCH GmbH. **c** Illustration of a molecular doping process facilitated by BF_4_^−^ anions. Reproduced with permission [64]. Copyright 2023, Wiley–VCH GmbH. **d** Schematic diagram illustrating how Me-4PACz fills vacancies and competes with DMSO to bind PbI_2_, promoting dissociation at the buried interface and enhancing perovskite crystallization to produce a denser layer. Reproduced with permission [65]. Copyright 2023, Royal Society of Chemistry

To regulate the residual PbI_2_ in perovskite films, several advanced methods have been developed with a focus on improving the film morphology, particularly at the grain boundaries through the use of precursor solution additives and post-processing treatments [78, 79]. Deng et al. argued that the modification of the buried interface is a key factor influencing both the quality of perovskite films and the presence of residual PbI_2_ [62]. They spin-coated 4,4′-diaminodiphenyl sulfone hydroiodide (DDSI_2_) onto the surface of SnO_2_ in order to modify the buried interface by finely regulating the distribution of residual PbI_2_ (See Fig. 2a). The − NH_3_^+^ and S = O functional groups in DDSI_2_ passivated defects in both the SnO_2_ and perovskite at the buried interface. DDSI_2_ also enhanced the wettability of SnO_2_ while establishing strong interactions, such as coordination and hydrogen bonding, with FAI and PbI_2_. This promoted the formation of high-quality perovskite films, refining the morphology and distribution of residual PbI_2_ at the interface. As a result of the passivation provided by PbI_2_ nanosheets, the champion PSC efficiency increased dramatically to 24.10%, with 93% of the performance retained after 1800 h of storage.

Compact TiO_2_ (c-TiO_2_), a commonly used ETL comparable in frequency to SnO_2_, has a relatively rough morphology that hinders perovskite nucleation and crystallization, resulting in lower crystallinity and greater nonradiative recombination, issues further compounded by the inherently lower electron mobility within c-TiO_2_ [80, 81]. Hence, innovative interfacial modifiers are required for the c-TiO_2_/perovskite junction to improve perovskite crystal formation, enhance charge-carrier dynamics at the interface, and reduce *V*_*oc*_ loss, thus improving overall device performance. Wang et al. utilized a 2D MXene as a passivator for the buried interface between CsPbI_3−x_Br_x_ and the ETL [63]. MXenes, which have the general formula M_n+1_X_n_T_x_, are transition metal carbides/carbonitrides known for their high conductivity, electron mobility, and thermal stability. The MXene Ti_3_C_2_T_x_, in particular, has been used to enhance electron mobility and charge transfer in SnO_2_ ETLs, improve perovskite crystallization and grain size, and serve as an interface passivator in PSCs to boost performance and stability [82–84]. MXenes, with their hydrophilic surface groups (O^2−^, OH^−^, and F^−^), have been shown to improve the wettability of the TiO_2_ surface and facilitate the rapid sublimation of DMAI, promoting the formation of CsPbI_3−x_Br_x_ perovskites [85–87]. MXenes can increase the interfacial energy, elevating the nucleation barrier, reducing nucleation sites, and promoting the growth of larger, high-crystallinity perovskite grains. This process enhances the film morphology, which has fewer defects and larger grain sizes. As illustrated in Fig. 2b, the MXene layer facilitates controlled nucleation, followed by slow crystal growth, allowing for the formation of high-quality perovskite films. This slow growth allows for Oswald ripening, where smaller particles dissolve and re-deposit on larger particles, leading to higher crystal quality and fewer defects [88].

When using semiconducting polymers as hole-transport materials in optoelectronic devices, many of their properties, including their carrier mobility, conductivity, WF, and energy levels, can be adjusted through doping or surface modification. They can also be readily fabricated into films and offer high light transmittance and strong electron-blocking ability. However, their intrinsically low carrier mobility and density tends to limit charge transfer at the HTL/perovskite interface, leading to charge recombination and energy loss (E_loss_) in p-i-n PSCs [64]. To enhance the carrier mobility and density of semiconducting polymers, strategies such as mixed doping and sequential doping have proven to be effective [89]. However, with mixed molecular doping, the dopant concentration needs to be carefully regulated because excess levels can compromise the quality and electronic performance of the polymer film, limiting its effectiveness [90]. In contrast, sequential doping, which involves either immersing polymer films in a dopant solution or vapor-depositing the dopant onto the surface of the polymer, mitigates the risk of excess dopant levels while preserving the film morphology [91]. For example, Luo et al. enhanced hole extraction in p-i-n PVSCs by sequentially doping PTAA with tris-(pentafluorophenyl)borane [92]. Nevertheless, this technique is hindered by the limited dopant penetration into the crystalline regions of the polymer [89]. To address this, Yamashita et al. introduced an anion-exchange strategy, which effectively increased doping levels in poly (2,5-bis(3-tetradecylthiophen-2-yl)thieno[3,2-b] thiophene) films [93].

Despite these advancements, further strategies are needed for p-i-n PVSCs to improve the electrical properties of HTLs and boost their charge-transfer efficiency. Zhang et al. used a novel molecular doping strategy in which BF_4_^−^ anion-assisted 1,3,4,5,7,8-hexafluorotetracyanonaphthoquinodimethane (F6TCNNQ) was introduced to improve the carrier mobility and density of PTAA and poly-TPD HTLs in p-i-n PSCs (Fig. 2c) [64]. This approach involved the exchange of BF_4_^−^ anions with F6TCNNQ^−^, which significantly enhanced the electrical properties of the polymer films and shifted their Fermi level (E_F_) closer to the highest occupied molecular orbital (HOMO) level. Additionally, the BF_4_^−^ anions interacted with the Pb^2+^ ions on the surface, improving the perovskite crystallinity, reducing V_I_, and converting the surface from an n-type to a p-type surface. This created a p-n homojunction, facilitating charge transfer and reducing energy level mismatches at the HTL/perovskite interface, thus lowering E_loss_. As a result, the optimized p-i-n PVSCs achieved a high PCE of 24.26% for PTAA and 22.65% for poly-TPD, along with enhanced stability. This strategy effectively boosted PVSC performance by improving charge transfer and minimizing energy offsets at the buried interface.

To overcome the efficiency limitations of single-junction cells, buried interface engineering has become a pivotal strategy in tandem PSCs. In particular, front wide-bandgap (WBG) PSCs generally experience a substantial V_OC_ deficit of approximately 0.5 V, which exceeds the 0.3 V deficit typically observed in conventional and narrow-bandgap cells [94, 95]. This higher V_OC_ deficit in WBG PSCs originates from three primary factors. Firstly, the interface between the WBG perovskite and charge selective layers often suffers from energy level misalignments and defects, resulting in greater non-radiative recombination [96]. Secondly, the rapid crystallization process of WBG perovskites tends to produce a high concentration of defects within the bulk material [97]. Lastly, the optoelectronic characteristics of the HTL significantly influence the crystallization process of the perovskite layer, thus affecting its overall quality and performance [65]. These factors combine to complicate efforts to optimize the efficiency of WBG PSCs. The most common HTLs in WBG PSCs include NiO_X_ and SAMs such as 2PACz and Me-4PACz. While NiO_X_ is attractive due to its stability, low cost, and optical properties, it suffers from significant defects, leading to V_OC_ deficits, while 2PACz only weakly adsorbs to the ITO surface and Me-4PACz exhibits poor perovskite coverage due to its high hydrophobicity [98]. To address these issues, various strategies, such as combining different HTLs or developing new materials, have been proposed, with one effective approach being the combination of NiO_X_ with SAMs to prevent direct contact between ITO and the perovskite, thus mitigating V_OC_ loss, enhancing hole extraction, and regulating perovskite crystallization [38, 99]. Cui et al. employed a method that uses the SAM 2PACz as the initial hole selective layer and subsequently modifies it with Me-4PACz (Fig. 2d**)**. Interface engineering with Me-4PACz was employed as a solution to the weak adsorption of 2PACz onto the ITO surface. While this poor adhesion leads to vacancies and uneven surface potential distribution, Me-4PACz filled these voids, creating a more uniform potential distribution across the substrate. In addition, the anti-solvent drop-casting process typically results in the accumulation of the dimethyl sulfoxide (DMSO)-PbI_2_ complex at the buried interface, which is exacerbated by subsequent thermal annealing that induces void formation. However, Me-4PACz altered this dynamic in Cui et al.’s study. Its phosphate group competed with DMSO for PbI_2_ coordination, accelerating the dissociation of DMSO-PbI_2_ complexes at the interface. Simultaneously, the synergistic interaction between Me-4PACz's hydrophobic methyl group and the hydrophilic phosphate group oriented the molecules with an upward phosphate configuration. As a result of this competitive coordination and molecular orientation, less DMSO remained at the buried interface, which facilitated crystallization and the formation of a dense perovskite layer. Some Me-4PACz molecules also accumulated on the surface in a disordered manner, further contributing to the interface properties. The combined effect of these processes (i.e., void filling, competitive coordination, molecular orientation, and surface accumulation) led to a more uniform potential distribution and a higher-quality perovskite layer. Therefore, a V_OC_ of 1.36 V in a PSC with a 1.78 eV bandgap was achieved, resulting in a champion PCE of 19.83%. This represents one of the lowest V_OC_ deficits (0.42 V) reported for a WBG PSC to date. This approach was also successfully implemented in tandem solar cell configurations. A two-terminal (2-T) all-perovskite tandem solar cell demonstrated an impressive efficiency of 27.34%, while a four-terminal (4-T) all-perovskite tandem solar cell achieved a champion efficiency of 28.05%. This demonstrated the significant improvements that Me-4PACz interface engineering can offer over traditional 2PACz approaches in terms of PSC fabrication.

## Impact of buried interfaces on long-term stability of PSCs

### Evaluating the thermal stability of buried interfaces: a multi-faceted investigative approach

**Fig. 3:**
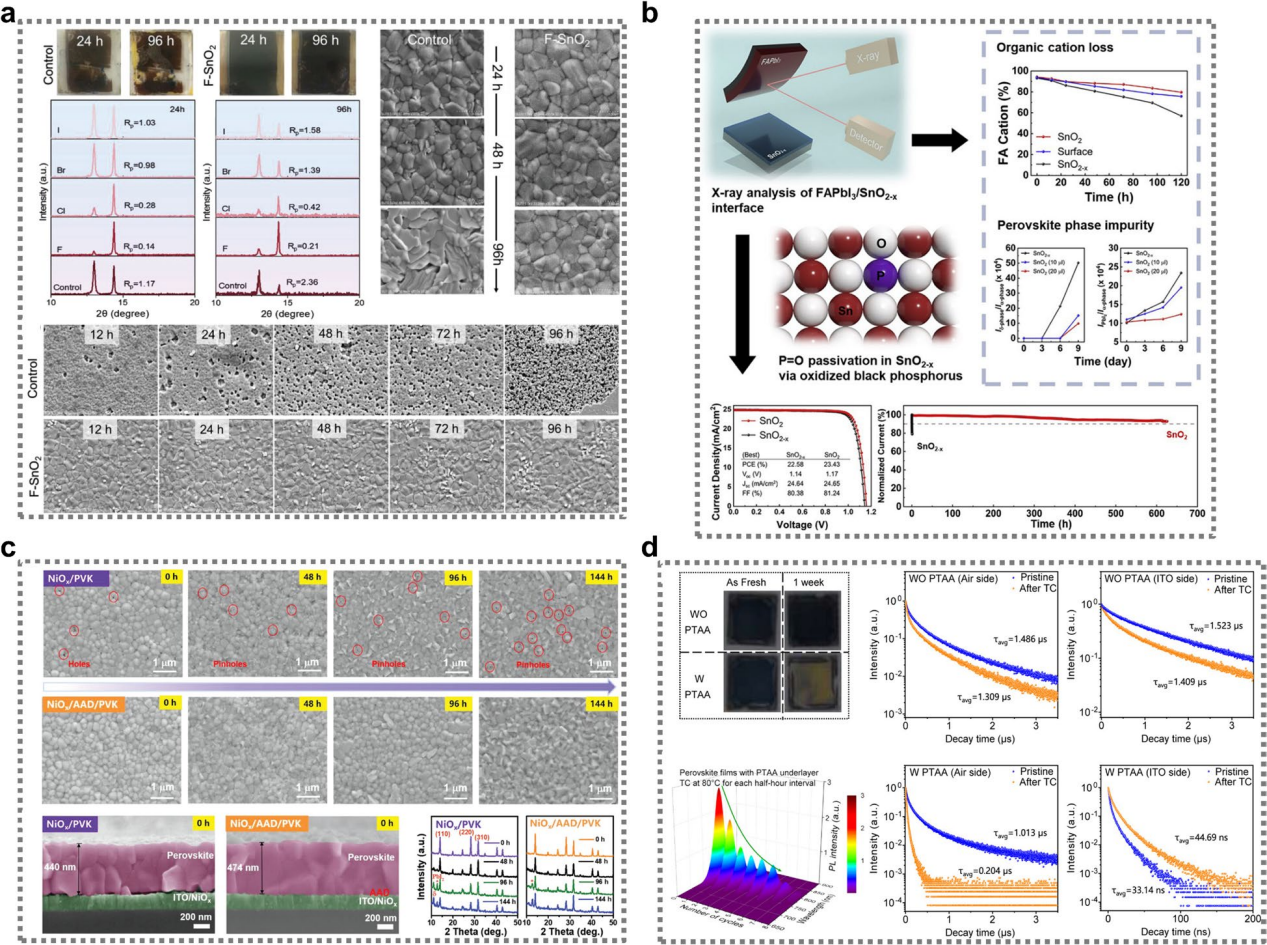
**a** Thermal stability of perovskite films at 85 °C and 35–45% relative humidity analyzed using images and XRD/SEM data, comparing control samples with F-SnO₂ after aging. Reproduced with permission [39]. Copyright 2023, Wiley–VCH GmbH. **b** Oxygen vacancies at the FAPbI₃/SnO₂ interface producing iodine interstitials, leading to a-FAPbI₃ degradation and reduced stability, which was mitigated using oxidized black phosphorus quantum dots, achieving a PCE of 23.23% with improved long-term and thermal stability. Reproduced with permission [100]. Copyright 2023, Elsevier Inc. **c** Time stability of NiOx/perovskite and NiOx/AAD/perovskite films stored in air at 85 °C analyzed using SEM images, cross-sectional SEM, and XRD measurements. Reproduced with permission [31]. Copyright 2023, Wiley–VCH GmbH. **d** Degradation of perovskite films over a week under 80 °C, comparing films with and without a PTAA underlayer through thermal treatment, in-situ PL decay, and TRPL measurements from both the air and ITO sides. Reproduced with permission [101]. Copyright 2024, Wiley–VCH GmbH

*XRD and SEM analysis.* The reliability of perovskite devices is heavily influenced by the thermal stability of perovskite films. Thus, there has been significant research interest in understanding how enhanced buried interfaces contribute to the morphological stability of these films. Thermal stability is typically examined using scanning electron microscopy (SEM) or surface images, which can be used to reveal morphological degradation, grain evolution, and other surface transformations caused by thermal stress. The thermal stability of perovskite films can also be assessed using X-ray diffraction (XRD) analysis, which provides insight into the crystallographic changes that occur under thermal stress. XRD patterns reveal how the crystalline structure of the perovskite evolves over time, including monitoring the emergence of decomposition products such as PbI_2_ and the transition between perovskite phases (e.g., from the α- to the δ-phase). By examining shifts in peak intensities and the appearance of new peaks, researchers can track the crystallinity, degradation, and phase segregation of perovskite films during thermal treatment, meaning that, along with SEM, XRD is a valuable tool for evaluating thermal stability. Figure 3a, c presents an example of the analysis of the thermal stability of perovskite films using SEM images and XRD spectra. This stability is strongly influenced by the buried interface, which mitigates degradation at high temperatures. Studies have shown that the quality and reactivity of buried interfaces, such as perovskite/SnO_2_ or perovskite/NiOx, directly affect the morphological and chemical stability of perovskite films during thermal treatment. With SnO_2_-based buried interfaces, thermal stress at 85 °C in ambient conditions led to severe degradation of the control perovskite film. This degradation was characterized by the formation of PbI_2_ flakes, as revealed by top-view SEM images, and the emergence of voids in the buried interface after just 12 h of heating (Fig. 3a). These voids were primarily caused by unfavorable reactions between the hydroxyl groups present on the SnO_2_ surface and perovskite cations, which acted as reactive centers and degraded the interface stability [39]. Over time, these voids increase, and after 96 h, the perovskite layer is fully decomposed at the interface. However, when fluorinated SnO_2_ (F-SnO_2_) is used as the buried interface layer, the thermal stability is greatly enhanced. The F-SnO_2_ layer promotes larger grain growth in the perovskite and reduces the formation of defects, resulting in significantly less morphological degradation and slower decomposition under the same thermal conditions. Similarly, when an amino acid derivative (AAD) is introduced as an interface modifier at the NiOx/perovskite buried interface, the stability of the perovskite film improves [31]. SEM and XRD analyses show that AAD-modified perovskite films exhibit larger and more uniform grains, with fewer pinholes and PbI_2_ crystallization after prolonged heating (Fig. 3b). The presence of AAD deactivates interfacial defects, optimizing the contact between NiOx and the perovskite layers, which delays the onset of degradation and phase segregation. Even after 144 h of thermal treatment, AAD-modified films show much less degradation compared to their unmodified counterparts, with the PbI_2_ peak appearing later and the δ-phase suppressed.

*XPS analysis.* Oxygen defects, such as oxygen vacancies in SnO₂, significantly impact the buried interface between perovskite and SnO₂, affecting the thermal stability and cation retention. Oxygen vacancies generate iodine interstitials at the FAPbI₃/SnO₂ interface, which the trigger degradation of the perovskite from its stable $\alpha$-phase to the undesirable $\delta$-phase and PbI₂. This leads to increased instability of the perovskite structure. The interface between FAPbI₃ and SnO_2-x_ was the focus of a systematic investigation by Lee and colleagues [100]. Their study revealed that oxygen vacancies on the SnO_2-x_ surface at this interface were responsible for triggering the formation of δ-phase FAPbI₃ and PbI₂. Seeking a solution to this, the researchers explored the use of a novel passivation agent. They discovered that oxidized black phosphorus quantum dots (O-BPs) could be effectively employed to mitigate these oxygen vacancies. The study focused on understanding the impact of thermal stress and oxygen vacancies in SnO₂ on the retention of organic cations (particularly FA) at the buried interface between perovskite and SnO₂, using X-ray photoelectron spectroscopy (XPS) measurements and analyzing the effects on PSC stability (Fig. 3b). FA cations, which are typically bound to the PbI₆ lattice via hydrogen bonding with iodine atoms, improve the stability of perovskite films. At the FAPbI₃/SnO₂ interface, the presence of well-defined oxygen atoms in SnO₂ provides additional hydrogen bonding sites for FA cations, which stabilizes the interface. However, when oxygen vacancies are present, these bonding sites are absent, resulting in higher cation loss. XPS measurements were used to monitor the loss of organic cations from the bottom surface of perovskite films under thermal stress. The perovskite films, deposited on SnO_2-x_ and subjected to annealing at 85 °C with a relative humidity of 15%, experienced significant FA cation loss. After 48 h of thermal treatment, the FA cation content in the perovskite layer exfoliated from SnO_2-x_ decreased significantly compared to that from SnO₂. Prolonged annealing for 72 h increased the loss to 57.03 mol % for SnO_2-x_-based films. In contrast, the perovskite film on SnO₂ exhibited higher cation retention, with the FA cation content only dropping by 6.07 mol %, illustrating the positive effect of reducing the number of oxygen vacancies on cation retention. The study also demonstrated that the absence of oxygen at the interface, combined with exposure to oxygen and moisture, exacerbates the loss of FA cations, especially in samples exposed to air. The retention of organic cations was directly linked to the operational stability of the PSCs, as illustrated by performance measurements. Devices with reduced oxygen vacancies in SnO₂ exhibited significantly higher thermal and operational stability. For example, after four days of heating, SnO_2-x_-based PSCs retained only 70% of their initial PCE, whereas SnO₂-based PSCs remained at 90%. Even when the HTL was switched to a thermally stable material (CuPC), the SnO_2-x_-based PSCs still exhibited a higher efficiency loss (10%) than the SnO₂-based devices (3.6%).

*PL analysis.* Shi et al. conducted a detailed investigation into the thermal stability of perovskite films, particularly focusing on the effect of the HTL on the degradation process [101]. FAPbI₃ films were coated onto substrates with and without a PTAA underlayer, and their degradation under moisture, heat, and solar illumination was compared. The study found that perovskite films with a PTAA underlayer degraded more quickly than bare perovskite films when subjected to thermal treatment at 80 °C. Significant degradation was observed in the PTAA-coated films, while the bare films remained relatively stable after one week. To understand this degradation, time-resolved photoluminescence (TRPL) measurements were taken. TRPL allowed the researchers to track the degradation of the upper interface of the perovskite (Fig. 3d). After thermal cycling, the perovskite films with PTAA demonstrated a sharp decrease in the photoluminescence (PL) lifetime, falling from 1 μs to 0.2 μs after one thermal cycle. In contrast, the bare perovskite films experienced only a slight reduction in the PL lifetime from 1.5 μs to 1.3 μs. Interestingly, the bottom interface of the PTAA-coated films exhibited a longer PL lifetime after thermal cycling, indicating the presence of ion migration at the interface between the perovskite and PTAA. This migration likely resulted in the accumulation of iodine ions, which created non-radiative recombination centers, further degrading the perovskite. The TRPL measurements, combined with this comparison between the top and bottom interfaces, identified the role of iodine ion migration in the accelerated degradation of perovskite films with a PTAA underlayer. This mechanism highlights the significant impact of the choice of the HTL on the thermal stability of perovskite films, emphasizing the need to optimize HTL materials to suppress ion migration and enhance device longevity.

Overall, these findings highlight the critical role of buried interfaces in dictating the thermal stability of perovskite films. By improving the quality of the buried interface—either through chemical passivation or the introduction of interface modifiers—thermal degradation can be significantly slowed, enhancing the longevity and reliability of perovskite-based devices under real-world operating conditions.

### Light-induced degradation at buried interfaces

**Fig. 4:**
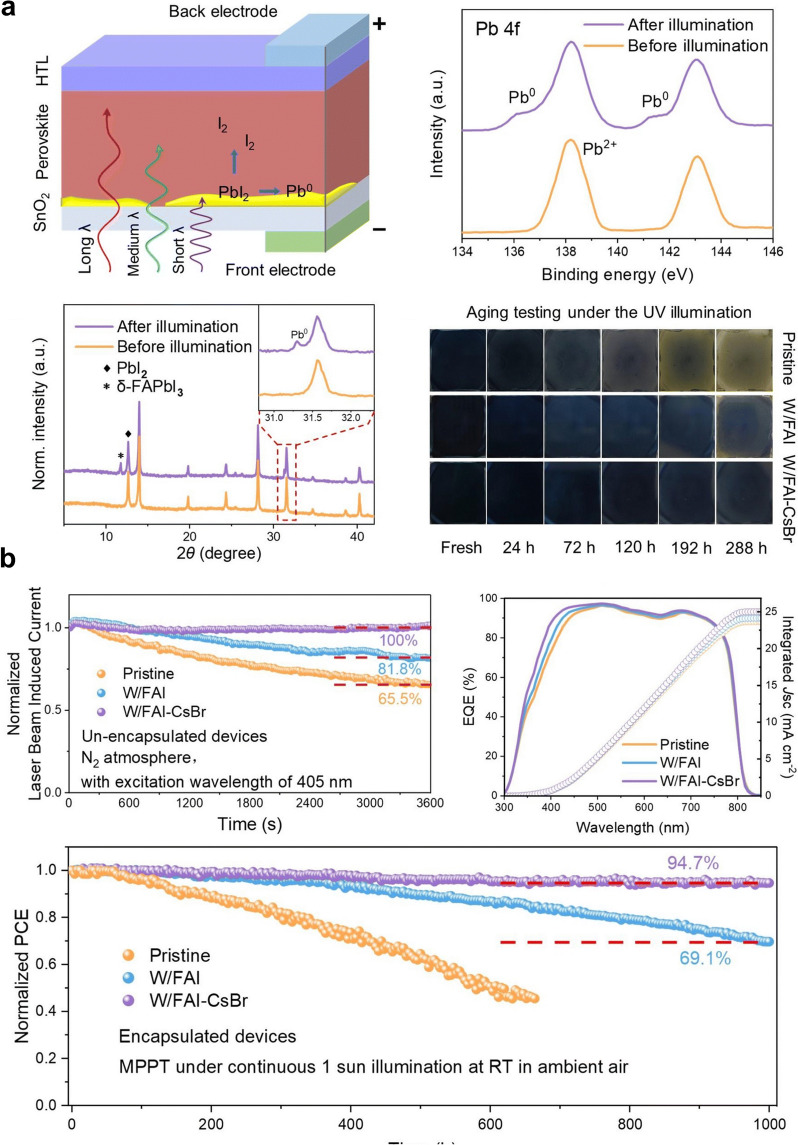
**a** Stability of perovskite films under UV light, including a schematic of solar cell illumination, XPS and XRD analysis after aging, and photographs of aged films in a nitrogen atmosphere. **b** LBIC evolution, EQE spectra, and integrated current density of PSCs, along with long-term stability results of encapsulated PSCs under continuous MPP tracking and LED illumination. Reproduced with permission [102]. Copyright 2023, Royal Society of Chemistry

The long-term stability of PSCs can be negatively impacted by the propensity of excess PbI₂ to degrade under light exposure, resulting in the formation of metallic lead (Pb⁰) and iodine (I₂) over time [103, 104]. Studies utilizing thermally and optically coupled quadrupole mass spectrometry have confirmed the photodecomposition of PbI₂ under illumination [105]. This degradation process is wavelength-dependent, with PbI₂ photodecomposition initiated by photon energies exceeding a threshold of 520 nm [102]. Given the limited penetration depth of shorter-wavelength light—such as 532 nm light, which penetrates approximately 100 nm [21] into a material—this decomposition predominantly affects regions near the illuminated interface, including the buried or bottom interface of PSCs. Consequently, controlling the levels of residual PbI₂, particularly at the lower interface of the perovskite layer, is essential for improving both the efficiency and long-term stability of PSC devices. As reported by Gao et al., the residual PbI₂ located at the buried interface underwent severe photodecomposition, which had a detrimental impact on the overall stability of the perovskite [102]. Under UV illumination, excess PbI₂ near the buried interface undergoes significant photodecomposition following the reaction PbI₂ → Pb⁰ + I₂ (g) PbI₂ → Pb⁰ + I₂ (g). The presence of metallic lead (Pb^0^) could be identified using XRD analysis with the observation of a subtle peak (Fig. 4a). This photodecomposition produces Pb⁰ and iodine (I₂), both of which contribute to device instability. Pb⁰ acts as a deep-level defect that increases non-radiative recombination and leads to the formation of Pb clusters, causing severe photo-instability, [106, 107] while I₂ catalyzes the degradation of adjacent perovskites, creating a feedback loop that generates more PbI₂ and I₂, increasing the internal pressure and producing pinholes or, in extreme cases, the fracturing of the top electrode of the device [108].

To address these issues, a pre-embedding mixed A-cation halide strategy was developed by Gao et al. [102]. By introducing a combination of formamidinium iodide (FAI) and CsBr into the SnO₂ layer, the residual PbI₂ at the buried interface was effectively transformed into a more stable 3D FA–cesium (FACs) perovskite. The success of this strategy was demonstrated in long-term stability tests. The target films, with pre-embedded A-cation halide salts, exhibited minimal degradation even after seven days of aging under UV light, in contrast to the pristine films, which exhibited numerous pinholes and degradation. XPS and XRD analyses also confirmed the absence of Pb⁰ peaks in the target films, emphasizing the effective stabilization of the perovskite material. Additionally, the W/FAI-CsBr films maintained their active α-phase for 288 h under continuous illumination, compared to only 72 h for the pristine films, which transitioned to the non-active δ-phase much earlier. The results clearly indicated improved phase stability in the target films, which was attributed to the effective suppression of I₂-induced phase transitions. The incorporation of FAI/CsBr into the SnO₂ layer resulted in a marked enhancement in device performance. Devices with pre-embedded mixed A-cation halides retained 94.7% of their initial efficiency after 1000 h of continuous illumination, a significant improvement over control devices (Fig. 4b). This longevity provided evidence that this strategy is effective in addressing both photodecomposition and phase instability, enhancing the potential use of PSCs in practical, long-term applications.

### Long-term stability of flexible devices

**Fig. 5:**
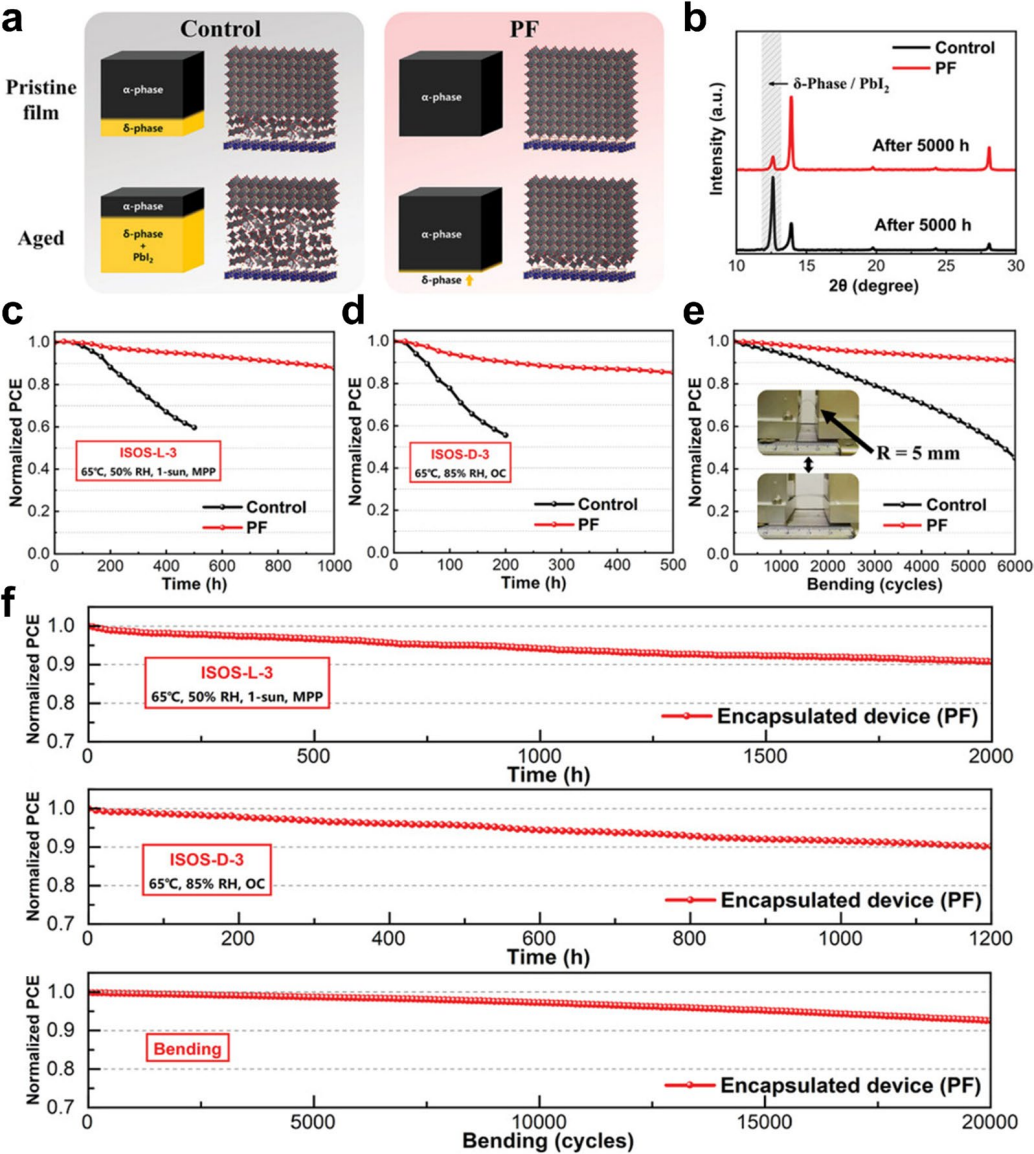
**a** Diagram showing the aging process of control and PF perovskite films, **b** XRD patterns of perovskite films after 5000 h of aging, **c** ISOS-L-3 and **d** ISOS-D-3 stability tests for various devices, **e** PCE variation according to the number of bending cycles (*R* = 5 mm), and **f** ISOS-L-3, ISOS-D-3, and bending stability tests for encapsulated devices. Reproduced with permission [109]. Copyright 2023, Wiley–VCH GmbH

In flexible PSCs, the buried interface is an important determinant of film quality and overall device performance [39]. This interface acts as the foundation for the crystalline active layer, significantly influencing the morphology, defect formation, and aging resistance of the perovskite film [110, 111]. Despite the importance of enhancing phase stability at the buried interface to increase the PCE and long-term stability, few studies have explored this. Xu et al. introduced proline hydrochloride (PF), a versatile mediator with multiple ammonium groups on a conjugated acridine backbone, to modify buried interface [109]. To explore the effect of PF passivation on device stability, they constructed an aging model for perovskite films. For unmodified control devices, rapid decomposition of the perovskite, marked by the formation of the δ-phase at decomposition sites, led to poor stability (Fig. 5a). In contrast, PF-passivated perovskite films maintained a pure α-phase with no visible decomposition sites, effectively retarding degradation (Fig. 5a). XRD analysis of aged perovskite films validated this model, revealing a significant reduction in δ-phase growth in PF-passivated devices (Fig. 5b). Moisture resistance, a key factor in device stability, was also greatly improved by PF modification. This innovative approach enables simultaneous passivation of interfacial defects and stabilization of the α-phase at the bottom of the FAPbI₃ layer, thus improving phase stability. The –NH₃⁺ groups in PF interact with iodide ions (I⁻) in the perovskite via hydrogen bonding, passivating surface defects and providing a stable environment for α-phase FAPbI₃ growth. Additionally, the PF layer, which is adsorbed in parallel between the SnO₂ and perovskite layers, offers enhanced coverage of the interface, further improving the passivation efficiency. Both the experimental results and DFT-based calculations confirmed that the growth of the undesired δ-phase of FAPbI₃ was significantly suppressed by the PF layer. This suppression was attributed to the longer distance and screening effect provided by the acridine ring, along with the ability of –NH₃⁺ groups to selectively interact with the SnO₂ layer, thereby promoting the growth of the desired α-phase FAPbI₃. As a result, the optimized PSCs achieved a remarkable PCE of 24.61% (certified at 23.51%). Furthermore, the PF-passivated devices demonstrated excellent durability, maintaining over 90% of their original PCE after 6000 bending cycles with a small bending radius of 5 mm, as well as exhibiting strong resistance to moisture and light. Rigorous testing based on International Summit on Organic Photovoltaic Stability (ISOS) protocols also confirmed the stability of the devices [112]. As shown in Fig. 5c–f, encapsulated flexible PSCs displayed outstanding storage stability, retaining 90.7% of their PCE after 2000 h (ISOS-D-3), impressive light-soaking stability, with 90.2% PCE retention after 1200 h (ISOS-L-3), and high mechanical durability, with 92.6% retention after 20,000 bending cycles [109].

## Characterization techniques for buried interfaces

The characterization of buried interfaces is used to understand device performance and stability. One of the primary techniques employed to investigate buried interfaces involves the careful removal of the perovskite layer from the substrate using epoxy. This process allows researchers to access and examine the lower surface of the perovskite, which is typically obscured during standard measurements. By peeling away the perovskite, it becomes possible to directly observe and analyze the interactions, defects, and chemical composition at the interface between the perovskite and the underlying substrate or charge transport layer. Because understanding the nature of the buried interface allows the performance and stability of perovskite-based devices to be optimized, ex-situ techniques have been employed as powerful tools for probing these hidden layers. Various advanced characterization methods, including XRD, atomic force microscopy (AFM), TRPL, XPS, SEM, transmission electron microscopy (TEM), and energy-dispersive X-ray spectroscopy (EDX) have been used to gain insights into the structural, morphological, and electronic properties of the underside of perovskite films. These techniques allow for a comprehensive assessment of the crystal orientation, defect distribution, surface roughness, and chemical composition, which are important in understanding how the buried interface influences overall device performance.

**Fig. 6:**
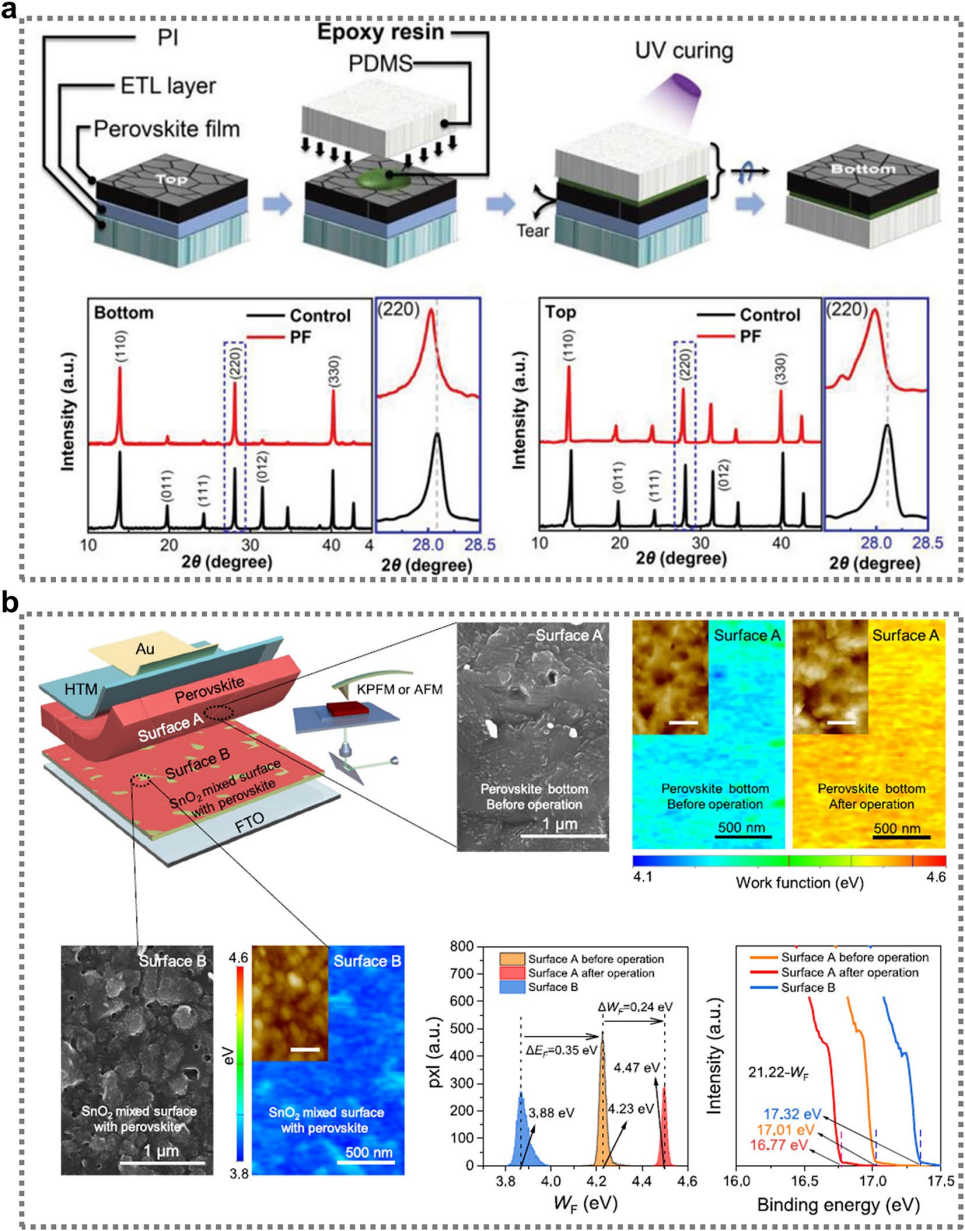
**a** Crystallization analysis involving the exfoliation of perovskite films followed by XRD analysis of the top and bottom layers to assess structural differences. Reproduced with permission [109]. Copyright 2023, Wiley‐VCH GmbH. **b** Peeling process for the buried interface and corresponding images and surface analysis before and after illumination using SEM, KPFM, and AFM for surfaces A and B, along with the WF distribution and UPS measurements. Reproduced with permission [66]. Copyright 2024, Elsevier Inc

The crystallization dynamics of the perovskite film, with particular emphasis on the bottom surface, were systematically investigated by Wu’s group to elucidate the effects of buried interface passivation [109]. In their study, they compared XRD data for both the top and bottom sides of perovskite films (Fig. 6a). The perovskite films were fabricated using SnO_2_ (as the control) and PF-modified SnO_2_. Prominent diffraction peaks at 13.91° and 28.07°, corresponding to the (110) and (220) planes, were visible in both the control and PF-modified films [113]. Notably, the bottom of the passivated films exhibited a trend where the peaks at (110) and (220) became dominant in the XRD spectra, indicating that the perovskite preferentially grew along these orientations. This suggests that the modification not only promoted more ordered crystal growth at the buried interface but also induced a shift in the (220) peak toward a lower angle in the PF-modified films, indicating a reduction in the stress within the perovskite layer as a result of PF treatment [113].

Zhang et al. employed a delamination technique to exfoliate the perovskite layer from a SnO_2_-functionalized FTO substrate, exposing the basal interface of the perovskite film for surface analysis (Fig. 6b) [66]. This technique allowed for a detailed examination of the basal surface of the perovskite film and its interaction with the SnO_2_-treated FTO substrate. The process began with the careful removal of the gold back electrode using adhesive tape, followed by a chlorobenzene wash to eliminate the HTL. Subsequently, the researchers applied a UV-curable adhesive to the perovskite layer and sandwiched it between the original substrate and clean FTO glass. After brief UV exposure, the solidified adhesive facilitated the separation of the perovskite film, revealing the underside of the perovskite (surface A) and the top of the SnO_2_ layer (surface B) [66]. Surface B had a faint grayish-brown hue, indicative of residual perovskite material. SEM confirmed this, revealing trace amounts of perovskite on the SnO_2_ surface. Kelvin probe force microscopy (KPFM) measurements of surface B yielded a WF of 3.88 eV, markedly lower than the 4.46 eV of pristine SnO_2_. This disparity suggested the formation of a weak adhesion zone within the perovskite layer itself, rather than at the SnO_2_ interface. This configuration could potentially enhance the long-term stability of the device.

In contrast, surface A displayed the characteristic roughness of pure perovskite, consistent with its known fragility. KPFM analysis of this surface revealed a WF of approximately 4.23 eV, a finding corroborated by UPS data. The significant WF difference between surfaces A and B indicated the presence of a homojunction with an inherent built-in potential (V_bi_), which could promote ion migration and defect accumulation [114]. Previous research has demonstrated that ion migration can induce shifts in the perovskite WF, with positive bias leading to more p-type characteristics [114]. To assess the dynamics of this interface, the researchers subjected PSCs from the same batch to 50 h of operation. Post-operation analysis revealed a shift in the WF of surface A to 4.47 eV, indicating ongoing ion migration. AFM revealed substantial changes in the surface morphology, with the roughness (Rq) increasing from 26.2 to 35.9 nm. This could be attributed to perovskite decomposition or phase changes resulting from extended light exposure. This pioneering approach provided unprecedented insights into the complex interactions at the buried interface of PSCs, offering a new perspective on device stability and performance optimization.

### Crystallization

The deposition of high-quality perovskite films onto HTLs in inverted PSCs is challenging. HTLs are involved in the formation of the perovskite absorber layer and its subsequent nucleation, yet their surface properties often hinder efficient film formation [116, 117]. Materials frequently used for HTLs, such as PTAA, NiOx, and SAMs, tend to exhibit either excessive hydrophobicity or instability when interacting with perovskite precursor solutions [117]. This mismatch prevents the formation of a uniform film, often resulting in defects at the buried perovskite–HTL interface. SAMs have emerged as promising candidates for HTLs in inverted PSCs due to their suitable energy levels for charge extraction and their potential to reduce non-radiative recombination [118]. However, SAMs such as [4-(3,6-dimethyl-9H-carbazol-9-yl)butyl] phosphonic acid (Me-4PACz) tend to distribute unevenly across the substrate and exhibit poor wettability for perovskite precursors [75]. This lack of uniformity complicates the deposition process, further contributing to the formation of defects that can reduce both the efficiency and stability of solar cells. Moreover, the solvents commonly used for processing perovskite films, such as N,N-dimethylformamide (DMF) and DMSO, often exacerbate these issues by interacting poorly with hydrophobic HTLs, thus generating additional morphological and compositional imperfections [119]. Consequently, simultaneous control over perovskite growth, defect passivation, and energy level alignment at the SAM-based buried interface remains challenging, emphasizing the importance of developing HTLs that can support defect-free, high-quality perovskite films with minimal voids and electronic traps to improve the performance and long-term reliability of inverted PSCs.

**Fig. 7:**
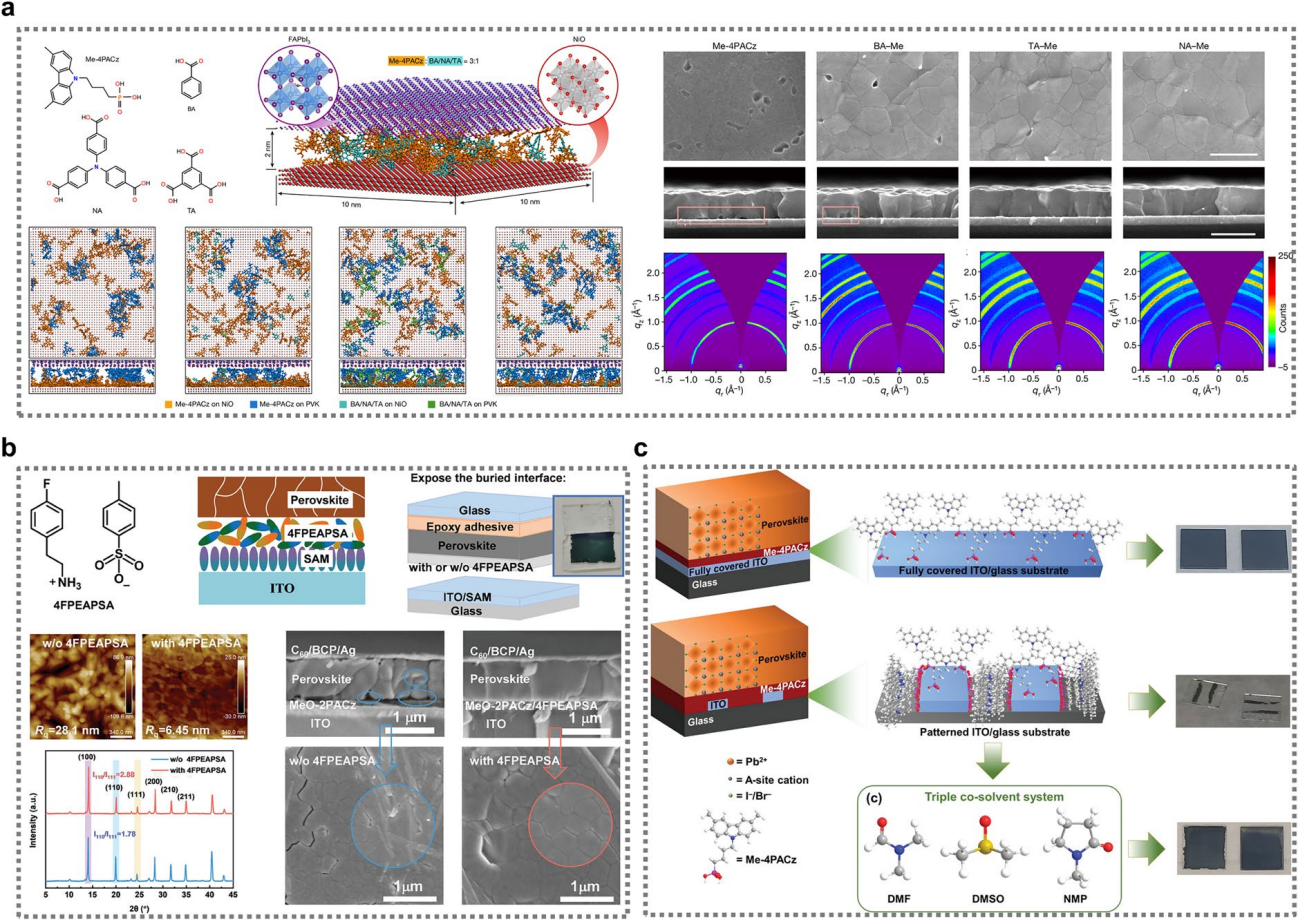
**a** Chemical structures of Me-4PACz, BA, NA, and TA, heterojunction model used for simulations, and molecular representations of perovskite/SAM/NiO heterojunctions (left image), and SEM images and GIWAXS patterns (right image) of perovskite films on the SAM hybrids Me-4PACz, BA–Me, NA–Me, and TA–Me. Reproduced with permission [71]. Copyright 2024, Springer Nature Limited. **b** Chemical structure of 4FPEAPSA, a simplified diagram of SAM and perovskite layers, a modified peeling-off method with images of the buried interface, AFM and SEM images of the exposed interface, and corresponding XRD curves. Reproduced with permission [41]. Copyright 2023, Wiley‐VCH GmbH.** c** Different assembly approaches of Me-4PACz and perovskite layers on ITO substrates, including uniform, disordered, and co-solvent-assisted strategies for achieving uniform perovskite coverage. Reproduced with permission [115]. Copyright 2023, Wiley‐VCH GmbH

To overcome these limitations, Liu et al. developed a hybrid SAM strategy in which Me-4PACz was combined with other functionalized molecules to form mixed SAM layers [71]. They found that combining Me-4PACz with 4,4′,4″-nitrilotribenzoic acid (NA) on NiO improved the surface wettability and reduced the agglomeration of Me-4PACz, facilitating the uniform deposition of the perovskite film. To elucidate the mechanisms involved in the hybrid SAM approach, the researchers conducted extensive theoretical screening and molecular dynamics simulations. Molecular models of the perovskite/SAM/NiO heterojunction were constructed, utilizing a 10 nm × 10 nm perovskite and NiO surface with a 2 nm layer of mixed SAMs at a 3:1 ratio of Me-4PACz to carboxylic acid molecules (Fig. 7a, left) [71]. The simulations revealed that, when used alone, Me-4PACz molecules tended to form dimers, trimers, and tetramers—an aggregation behavior consistent with previous reports [75] that leads to the formation of undesirable nanovoids at the bottom of the perovskite film. While the addition of benzoic acid (BA) had limited success in reducing this aggregation and offered little control over nanovoid formation, the incorporation of NA or trimesic acid (TA) into the Me-4PACz layer produced significantly better results. Both NA and TA created a more compact hybrid HTL, suggesting that the molecular structure and functionality of the carboxylic acid compounds play a key role in modulating the self-assembly process and film morphology. Introducing NA molecules into the Me-4PACz layer notably improved the wettability of the perovskite solution, reducing nanovoids and relieving residual stress at the interface. This hybrid SAM configuration also minimized the agglomeration of Me-4PACz, resulting in a more uniform distribution across the NiO surface, thus improving the charge extraction efficiency and reducing non-radiative recombination loss. These findings were supported by theoretical modeling and molecular dynamics simulations, which revealed that NA created a compact and homogeneous HTL structure, resulting in enhanced interface properties. Experimental validation using advanced characterization techniques such as SEM and grazing-incidence wide-angle X-ray scattering (GIWAXS) confirmed that perovskite films deposited on NA–Me SAMs exhibited a smoother and more compact morphology with enhanced crystallinity (Fig. 7a, right). Furthermore, the hybrid SAMs effectively relieved tensile stress at the buried interface, contributing to the improved performance and stability of the PSCs.

Other approaches to buried interface passivation were investigated by the Tan [41] and Wu [120] groups. Notably, the Tan group used an amphoteric organic salt (4FPEAPSA) to modify the SAM layers. This approach, which was supported by DFT calculations, significantly improved the crystallinity and morphology of the perovskite films at the buried interface [41]. SEM analysis revealed that the 4FPEAPSA-treated films had a smoother and more compact structure, with larger grain sizes compared to untreated films (Fig. 7b). The reduction in nanovoids and cracks at the buried interface led to more uniform crystal growth. XRD results confirmed the improved crystallinity, with an increase in the intensity of the < 100 > facet and a decrease in the full width at half maximum (FWHM), indicating fewer defects and an enhanced crystal orientation. The Wu group improved perovskite film formation by using MPA-CPA as an amphiphilic hole transporter on PTAA films, enhancing wettability and layer compatibility [120]. MPA-CPA's hydrophilic and hydrophobic components allowed better interaction with both the perovskite and the electrode, leading to a more uniform, void-free perovskite layer compared to PTAA and 2PACz. Advanced microscopy revealed seamless contact between the perovskite and ITO on MPA-CPA, reducing recombination and improving performance, highlighting its potential to enhance PCE and stability in perovskite solar cells.

Kulkarni et al. introduced a novel triple co-solvent system comprising DMF, DMSO, and N-Methyl-2-pyrrolidone (NMP), enhancing the interaction between the perovskite ink and the Me-4PACz-coated substrate [115]. The inclusion of NMP, a slightly nonpolar component, is hypothesized to improve interaction with Me-4PACz. They initially encountered challenges in forming uniform perovskite layers. Water contact angle measurements revealed Me-4PACz's hydrophobic nature (85°), attributed to its methyl (─CH_3_) and long alkyl chain (C_4_H_8_) groups. Comparatively, MeO-2PACz showed better wettability due to its less hydrophobic methoxy (─OCH_3_) group and shorter alkyl chain (C_2_H_4_). They observed that Me-4PACz forms a uniform vertical assembly on fully covered ITO substrates, resulting in a uniform perovskite layer. However, on patterned ITO substrates, perovskite formation was limited to the ITO regions, consistent with previous findings [33]. The group proposed that horizontal self-assembly of Me-4PACz occurs on the vertical sides of patterned ITO, creating a dense monolayer extended by the long alkyl chain. The use of ethanol, a highly polar solvent, for Me-4PACz dissolution may hinder SAM formation on glass surfaces, as suggested by earlier studies [115]. This could explain the non-uniform Me-4PACz orientation on glass parts of patterned ITO substrates, leading to poor perovskite ink interaction and irregular layer formation. The triple co-solvent strategy significantly improves perovskite coverage on Me-4PACz SAM deposited on patterned ITO substrates as shown in Fig. 7c. This approach not only enhances film uniformity but also addresses the challenges associated with large-area device fabrication and diverse perovskite compositions.

### Band alignment

**Fig. 8:**
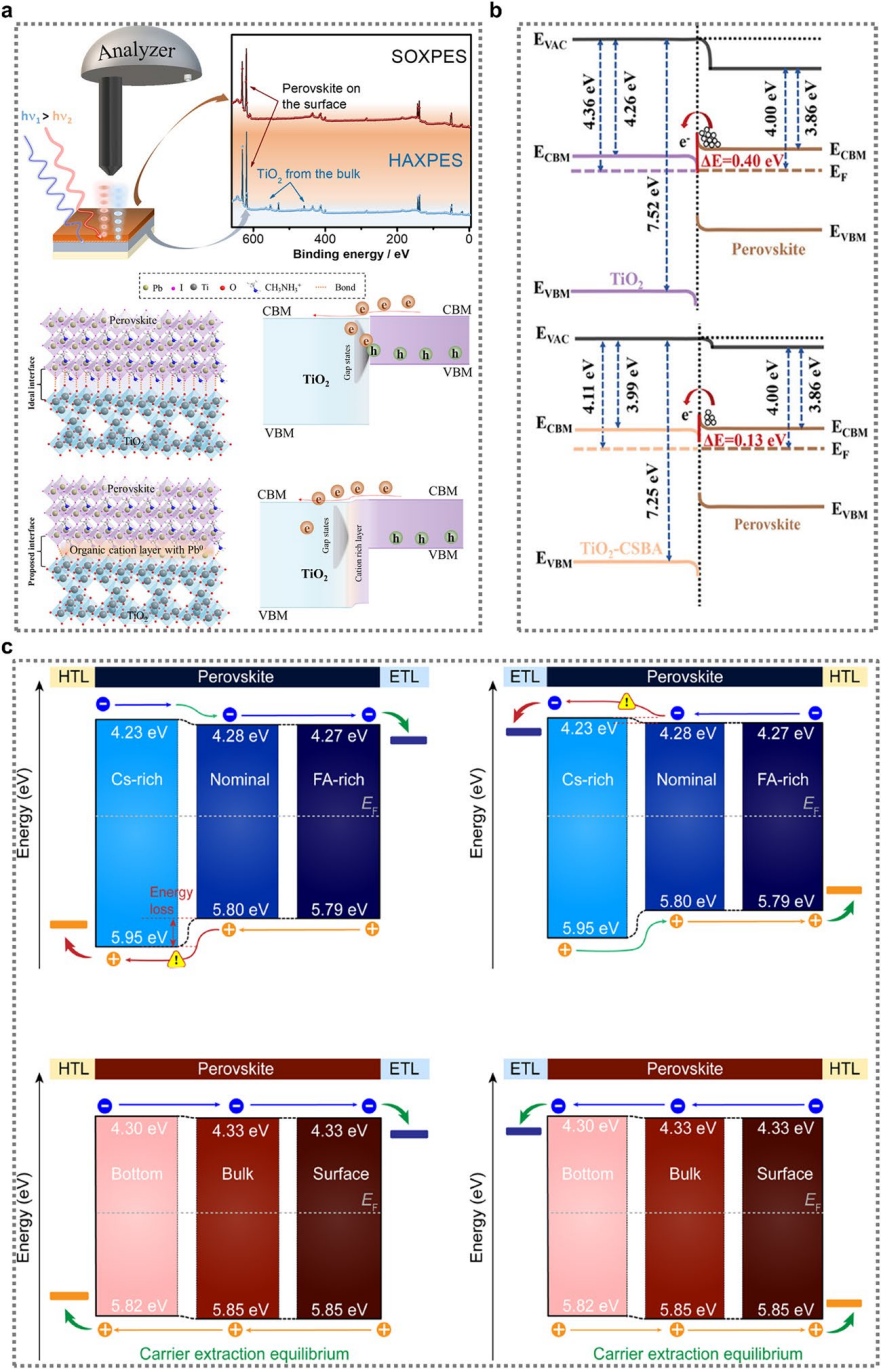
**a** Setup and survey spectra from the SOXPES and HAXPES analysis of TiO_2_/perovskite and theoretical and experimentally verified TiO_2_/perovskite interface properties, including Pb attachment to Ti − O, and corresponding band diagrams. Reproduced with permission [121]. Copyright 2023, American Chemical Society. **b** Energy diagrams of the TiO_2_/perovskite interface (above) and the TiO_2_-CSBA/perovskite interface (below). Reproduced with permission [122]. Copyright 2024, Wiley–VCH GmbH.** c** Schematics for the band alignment of the p-i-n structure**.** Reproduced with permission [20]. Copyright 2023, Springer Nature

Photoelectron spectroscopy (PES) is an in-situ analytical technique widely used to assess buried interfaces in electronic devices. It is employed to directly examine chemical reactions on the surface and at the interface of materials, providing crucial insights into electronic properties and band alignment. PES is thus particularly valuable for understanding and optimizing interfaces in devices such as PSCs, where interface characteristics strongly impact overall performance [123]. For example, a study by Olthof and colleagues demonstrated the close connection between the initial stages of perovskite growth and the surface chemistry of the underlying substrate, [124] highlighting the impact that interfacial band edge positions can have on PSC performance. Similarly, Das et al. investigated the chemical composition and electronic characteristics of the interface between a TiO_2_ ETL and a perovskite using advanced X-ray PES techniques [121]. To develop a comprehensive profile of the interface, perovskite layers of varying thicknesses were prepared by spin-coating diluted perovskite solutions onto TiO_2_ films. The concentrations used were 0.2 M, 0.5 M, and 1.2 M, resulting in approximate layer thicknesses of 5 nm, 15 nm, and 400 nm respectively. They employed a dual-technique strategy that used surface-sensitive soft X-ray photoelectron spectroscopy (SOXPES) to probe the uppermost layers and hard X-ray photoelectron spectroscopy (HAXPES) to explore the bulk properties and deeper interfaces. The results revealed beneficial upward band bending in the TiO_2_ layer when in contact with the spin-coated perovskite. Another key finding was the formation of an organic-rich interfacial region within the topmost nanometer of TiO_2_, which appeared to play a role in passivating surface defects (Fig. 8a) [121]. This layer was characterized by complex chemical interactions, with oxygen from TiO_2_ forming bonds with organic cations and partially with [PbI_6_]^4−^ complexes, while titanium interacted with iodine from the perovskite structure. Notably, they observed the presence of unreacted Pb^0^ within this organic-rich zone. Valence band edge analysis utilizing resonance PES combined with X-ray absorption spectroscopy suggested that the TiO_2_ at the perovskite interface had fewer defects compared to its bare counterpart. This indicated that the spin-coating process itself may serve as a defect passivation mechanism, potentially eliminating the need for additional intermediate layers. Current PSCs utilizing this interface without modifications achieve PCEs of around 18%, supporting the self-passivation hypothesis for TiO_2_-related interface defects [121]. However, the Pb^0^ detected at the interface could potentially limit device performance by reducing the photovoltage. Therefore, surface modification techniques or altering precursor chemistry should be explored to mitigate Pb^0^ formation. Introducing an interlayer might also enhance long-term PSC stability by preventing direct TiO_2_–perovskite interactions, which could otherwise lead to interface degradation under UV exposure.

One particularly effective method for modifying the buried interface in PSCs is the implementation of molecular bridges, which have shown promise in enhancing carrier transport and perovskite crystallization at the interface [122]. Researchers have explored different compounds to create these molecular bridges. For example, a group led by Peng utilized 3-amino-4-pyrazolecarboxylic acid (APA) to address interfacial defects and improve carrier movement, resulting in PSCs with impressive efficiency [125]. Similarly, Xu and colleagues employed 3-sulphonato-propyl acrylate potassium salt (SPA) at the TiO_2_/perovskite interface, leading to significant improvements in CsPbI_3_-based PSCs [83]. Huang et al. also implemented a ligand-engineered deposition strategy using additive ligands such as tartaric acid (TA), which can passivate surface oxygen vacancies by binding to Ti atoms, and cross-link with perovskite films through interactions with Pb atoms via the –COOH group [126]. Selecting suitable organic molecules is essential for designing molecular bridges because molecules with multiple coordination groups can link metal atoms in both the ETL and perovskite.

While various molecules have been used to construct molecular bridges, little attention has been paid to their arrangement orientation at the buried interface. Compounds such as APA, SPA, TA, and α-cyano-4-hydroxycinnamic acid (CHCA) have been employed successfully, but their molecular orientation at the interface is often assumed to be random. However, there is growing recognition that an oriented molecular bridge could potentially enhance energy alignment at the heterointerface and create a more uniform environment [127]. Consequently, developing strategies to construct oriented molecular bridges may be key to maximizing the effectiveness of interface bridge techniques in boosting PSC efficiency. Wang et al. [122] explored 4-chloro-3-sulfamoylbenzoic acid (CSBA) as an oriented molecular link at a buried interface, demonstrating that its strategic introduction to the TiO_2_ surface prior to perovskite deposition facilitated a precise, directional alignment of the CSBA molecules. Figure 8b presents a conceptual representation of the energy band curvature at the TiO_2_/perovskite junction. A significant finding from this analysis was the reduction in the energy level discrepancy between the two materials. Specifically, the difference between the conduction band minima of the perovskite and TiO_2_ (denoted as ΔE) decreased from 0.40 eV to only 0.13 eV. This marked improvement in the energy level alignment can be attributed to the introduction of the oriented CSBA molecular bridge at the interface [122]. The presence of this strategically arranged molecular layer appeared to facilitate the more efficient transfer of electrons across the TiO_2_/perovskite boundary. Thus, TiO_2_-based planar PSCs with an active area of 0.08 cm^2^ achieved a certified PCE of 25.32%, the highest reported to date, while large-area (1 cm^2^) PSCs reached a PCE of 24.20% [122]. Additionally, non-encapsulated PSCs exhibited excellent long-term stability, retaining approximately 91% and 85% of their initial PCE after 3000 h under ambient conditions and 1200 h under UV exposure, respectively.

The Pan group conducted a comprehensive analysis of internal energy levels using advanced UPS to investigate variation in the composition of the perovskite FA_0.95_Cs_0.05_PbI_3_ with depth [20]. Their findings revealed the formation of a quasi-type I energy band configuration near the Cs-rich region, extending over several hundred nanometers. This configuration manifested as a downward shift in the lowest unoccupied energy state and an upward shift in the highest occupied energy state (Fig. 8c) [20]. This arrangement can negatively impact charge carrier movement in solar devices, regardless of whether they employ a p-i-n or n-i-p structure. This effect is due to electrical doping phenomena [128]. The consequence of this band misalignment is a significant imbalance in electron and hole extraction efficiency, which can severely compromise device performance, with the fill factor particularly susceptible to degradation. However, the introduction of a buried interface passivator transformed the band diagram into a more favorable, flattened configuration, which effectively minimized the energy losses associated with charge carrier transport within the perovskite layers.

### Advanced strategies for achieving homogeneity in buried interfaces

**Fig. 9:**
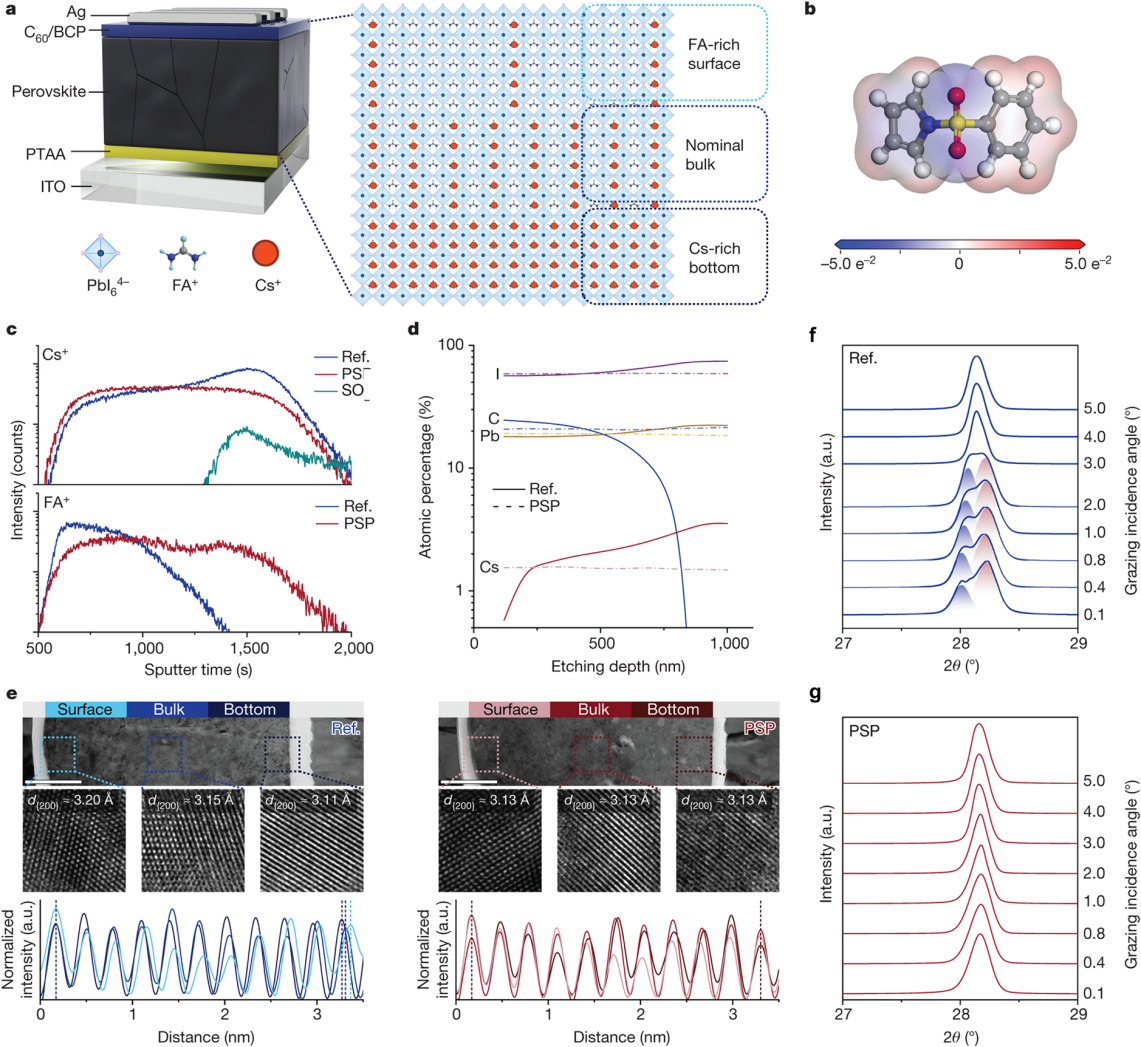
**a** Inhomogeneous phase distribution due to FA and Cs segregation and the cation distribution. **b** Electrostatic potential image and molecular structure of PSP. **c** ToF–SIMS and **d** XPS data showing the cation distribution and atomic percentages in reference and PSP devices. **e** High-angle annular dark-field TEM images of both reference and PSP-treated samples, with high-resolution TEM displaying lattice structures (inset). GIXRD spectra showing phase segregation, highlighting Cs-rich and FA-rich regions in **f** reference and **g** PSP-treated films. Reproduced with permission [20]. Copyright 2023, Springer Nature

Achieving uniformity in PSCs has become a central focus of recent research, particularly in terms of the distribution of cations within the material. While the inclusion of FA and cesium (Cs) at the A-site of perovskites has shown promise as a means to enhance efficiency, [129] it has also raised concerns about long-term stability due to potential cation segregation [130]. Analysis of the spatial distribution of these cations has revealed their tendency for non-uniform arrangement, especially in the out-of-plane direction (Fig. 9a) [20]. This out-of-plane cation inhomogeneity has been observed by Liang et al. to have a profound impact on device performance. To overcome this challenge, they introduced 1-(phenylsulfonyl) pyrrole (PSP) as an additive to mitigate cation segregation in FA-Cs perovskites (Fig. 9b) [20]. This approach led to the development of p-i-n structured devices with a champion PCE of 26.1% (with certified values of 25.8% for the reverse PCE and 25.2% for the steady-state PCE). Studies utilizing advanced analytical techniques such as time-of-flight secondary-ion mass spectroscopy (ToF–SIMS) (Fig. 9c) and XPS (Fig. 9d) have provided compelling evidence of this non-uniformity. These methods have revealed a gradient in cation distribution, with Cs congregating near the bottom of the perovskite film, while FA follows the opposite trend.

To visualize the A-site cation distribution and its effects on lattice uniformity, Liang et al. employed cross-sectional TEM to examine the surface, bulk, and bottom layers of the perovskite film (Fig. 9e). By measuring the interplanar spacing (d) of the lattice in these areas, direct evidence was gained into the phase heterogeneity along the depth of the film. The observations revealed a clear gradient in the {200} plane spacing (d{200}) for the untreated reference film, with measured values of 3.20 Å at the surface, 3.15 Å in the bulk, and 3.11 Å at the bottom. The decreasing trend in d values from the top to bottom indicated increasing internal lattice stress within the film. The notably smaller d_bottom_ suggested a significant lattice mismatch [131] at the film's base, likely due to the accumulation of smaller Cs atoms forming a Cs-rich perovskite phase. This observed lattice contraction provides strong evidence that cation inhomogeneity contributes significantly to lattice strain [132]. These structural inconsistencies can have severe effects on the material's optoelectronic properties and overall device performance.

This inhomogeneity also influences the crystal structure of the perovskite. Grazing incident X-ray diffraction (GIXRD) analysis also uncovered the presence of a Cs-rich phase near the bottom interface, indicated by shoulder peaks in the diffraction patterns (Fig. 9f) [133]. The structural irregularities were notably reduced with the introduction of PSP (Fig. 9g). The out-of-plane compositional inhomogeneity originated from the segregation of FA and Cs phases during film formation, even at low Cs concentrations. This segregation resulted in a gradient structure, with an FA-rich phase at the top, a nominally stoichiometric middle region, and a Cs-rich phase at the bottom (Fig. 9a) [20]. The addition of PSP proved effective in promoting a more homogeneous cation distribution throughout the perovskite film. As expected, the increased uniformity improved both the stability and performance of PSCs by reducing the lattice strain and mitigating the formation of undesired Cs-rich regions.

**Fig. 10:**
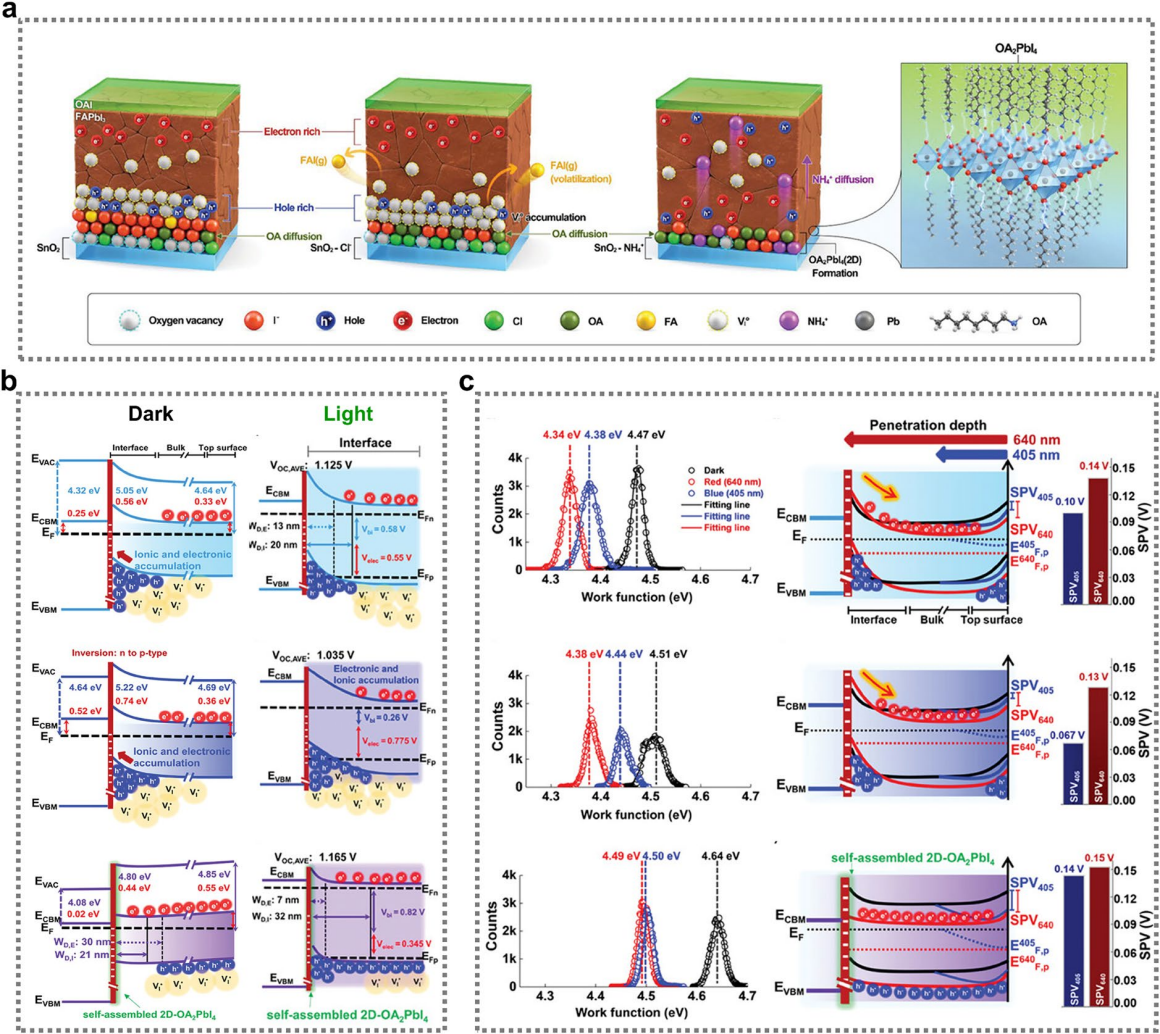
**a** Charge distribution and ionic dynamics at the perovskite interfaces with different ETLs and the emergence of self-assembled 2D-OA_2_PbI_4_. **b** Overall band diagrams for FAPbI_3_ on various ETLs constructed using UPS and DC polarization measurements in the dark, with interface band diagrams measured under light illumination. **c** WF values under dark and illuminated conditions obtained using KPFM, and corresponding band diagrams constructed from the surface photovoltage results for SnO_2_, Cl^−^-SnO_2_, and NH_4_^+^-SnO_2_ interfaces. Reproduced with permission [21]. Copyright 2024, Wiley–VCH GmbH

The dual electronic and ionic conductivity of halide-based perovskites, driven by iodine vacancies, can cause stoichiometric imbalances [134]. These imbalances lead to the degradation of narrow-bandgap compositions and phase separation in wide-bandgap perovskites, reducing the charge extraction efficiency by increasing the non-radiative recombination [135]. To address this issue, various interface engineering techniques, such as the use of additives and passivation, have been explored. However, conventional passivation materials can cause electronic charge accumulation and non-uniformities, leading to performance issues [53]. Given the complexity of analyzing the relationship between heterojunction interfaces, passivation materials, and their impact on perovskites, a stronger understanding of charge carrier dynamics is crucial. In line with this, Kim et al. investigated the SnO_2_/FAPbI_3_ buried interface in PSCs with an ITO/SnO2/FAPbI3/OAI/Spiro-OMeTAD/Au configuration, incorporating SnO_2_, Cl^−^-SnO_2_, and NH_4_^+^-SnO_2_ as ETLs [21]. Interestingly, they discovered that a self-assembled 2D perovskite formed at the NH_4_^+^-SnO_2_/FAPbI_3_ buried interface, where the low migration barrier of NH_4_^+^ facilitated its substitution by OA^+^, resulting in the formation of 2D-OA_2_PbI_4_. The self-assembled 2D-OA_2_PbI_4_ interface inhibited ionic charge accumulation, reduced iodine vacancies, and increased resistance to iodide oxidation. This resulted in lower ionic conductivity and higher electronic conductivity under illumination, promoting a uniform electronic charge distribution throughout the perovskite film (Fig. 10a). Under illumination, all samples exhibited upward band bending due to hole accumulation at the negatively charged interface. In the SnO_2_ and NH_4_^+^-SnO_2_ samples, depletion effects were observed, while the Cl^−^-SnO_2_ sample exhibited both electronic and ionic accumulation. The V_OC_ of Cl^−^-SnO_2_ was maintained through hole accumulation, unlike in NH_4_^+^-SnO_2_. Iodide segregation led to significant V_I_ accumulation and a smaller ionic depletion region. At the 2D perovskite self-passivated interface, increased resistance to iodide oxidation was noted, with lower ionic conductivity and higher electronic conductivity under illumination.

Following the formation of 2D-OA_2_PbI_4_ structures, excess NH_4_^+^ infiltrated the FAPbI_3_ layers. These ions tended to occupy vacancies associated with FA^+^ cations and Pb^2+^ ions. This infiltration process induced a significant change in the WF of the material. The WF at this interface (4.8 eV) was lower than at the film surface (4.85 eV), in contrast to observations for the SnO_2_ and Cl^−^-SnO_2_-based devices. This change in the WF led to an optimized band-bending configuration, which was observed both at the interfacial region and in the film's upper surface This favorable energy level alignment in NH_4_^+^-SnO_2_-based devices was expected to enhance electron extraction at the ETL/perovskite interface and hole extraction at the perovskite/HTL interface. The lower WF of NH_4_^+^–SnO_2_ also led to an increased built-in potential, potentially improving charge separation and collection within the device (Fig. 10b).

To investigate charge carrier distribution in PSCs, surface photovoltage (SPV) measurements, assisted by KPFM, were used. Using lasers with different penetration depths (640 nm and 405 nm), the study examined charge behavior across various regions of the perovskite film, including the interface, bulk, and surface (Fig. 10c). The 2D perovskite was found to have a key role in passivation. The study demonstrated that 2D perovskite passivation effectively mitigated both surface and bulk defects in the perovskite films. This passivation strategy led to a more homogeneous charge distribution across the perovskite films under illuminated conditions. The effectiveness of 2D perovskite passivation was particularly evident in the NH_4_^+^–SnO_2_ sample, which exhibited the highest SPV at 640 nm (SPV_640_) of the ETLs tested [21]. This high SPV_640_ correlated with the higher V_OC_ observed for this configuration. By facilitating a more uniform charge distribution and reducing defects, 2D perovskite passivation potentially enhanced the overall optoelectronic performance of the material. This improvement in charge dynamics and reduced recombination loss could lead to more efficient PSCs. The 2D perovskite assisted in optimizing the interface and bulk properties of the perovskite film, contributing to greater charge separation and transport, which are key factors in solar cell performance.

## Summary and outlook

PSCs have improved dramatically in terms of their PCE and now represent a promising alternative to traditional photovoltaic technologies. Researchers have comprehensively examined the critical role of buried interfaces in both n-i-p and p-i-n PSC architectures, with a focus on how these interfaces influence charge extraction, recombination, and device stability. Through detailed analysis, they have explored the importance of material selection for ETLs and HTLs and the impact of defect passivation strategies on overall device performance. Particularly, the introduction of SAMs as interface modifiers has led to significant improvements in reducing recombination loss and achieving optimal energy level alignment. Additive engineering, combined with the development of novel passivation layers, has further enhanced the quality of perovskite films by minimizing defects and improving crystallization. Additionally, advanced techniques such as DFT modeling and experimental analysis have provided more detailed insights into how buried interfaces control the efficiency and stability of PSCs. The fine-tuning of these interfaces is crucial for addressing the long-standing challenges of PSCs, particularly improving device stability under operational stress, scalability, and long-term performance. The insights gained from buried interface engineering can accelerate the development of PSCs for real-world applications.

Despite the significant advancements in understanding and optimizing buried interfaces in PSCs, several challenges remain. The issue of long-term stability, particularly in response to humidity, heat, and light exposure, requires further research. Future studies should continue to explore novel interface materials and advanced passivation techniques that can suppress degradation pathways at buried interfaces. Moreover, improving the scalability of these techniques for industrial production is required to ensure the commercial viability of PSCs. Emerging approaches, such as tandem solar cells and multi-junction designs, hold promise for pushing the efficiency limits of PSCs beyond current benchmarks. In these designs, interface engineering will play a pivotal role in mitigating energy loss and ensuring seamless charge transport across different layers. Additionally, machine learning and artificial intelligence-driven approaches to material discovery and interface optimization could accelerate the development of high-performance PSCs. In conclusion, while buried interface engineering has brought PSCs closer to their theoretical efficiency and stability limits, continued innovation in materials science and device architecture is essential to fully realize their potential in sustainable energy applications. The next phase of research should focus on integrating these advances into large-scale manufacturing processes to promote the use of PSCs as a next-generation photovoltaic technology.

## Data Availability

Not applicable.
